# Deep Gamification and Artificial Intelligence as Catalysts of Educational Transformation

**DOI:** 10.12688/f1000research.171453.1

**Published:** 2025-10-22

**Authors:** Diana Milena Patiño Barriga, Ana Dolores Vargas Sánchez, Paloma Valdivia Vizarreta

**Affiliations:** 1 Universidad de La Sabana, Chia, Cundinamarca, Colombia; 2 Universitat Autonoma de Barcelona, Barcelona, Cataluña, Spain

**Keywords:** Gamification, artificial intelligence, learning, innovation, social transformation

## Abstract

This opinion article examines the convergence between artificial intelligence (AI) and gamification in learning environments, with an emphasis on deep gamification designs aimed at creating interactive and meaningful experiences that transcend extrinsic motivation. The introduction sets the context by presenting AI as a tool that must be critically analyzed within the framework of critical pedagogy, which underscores the importance of adopting technology reflectively, placing the student at the center, and responding to the real needs of the community. The body of the article develops this claim: the integration of artificial intelligence into deep gamification designs can contribute to genuine educational transformation, provided that such integration is guided by critical pedagogical principles and maintains a balance between the potential of technology and the formative power of teaching practices. Arguments related to this claim are provided, including the use of AI in gamified designs to create personalized learning experiences based on students’ needs, rhythms, and learning styles; to transform the way students learn; to offer new educational resources; to adapt elements such as difficulty levels, challenges, and feedback in real time; and to develop engaging learning systems that lead to better academic outcomes. The conclusion emphasizes the significance of instructors utilizing critical pedagogy to direct AI-optimized gamification designs by incorporating culture, creativity, and critical awareness as pillars of educational transformation. This approach enables the surmounting of the obstacles presented by this synergy.

## Introduction

In the 21st century, the integration of digital technologies into modern education has been characterized by narratives advanced by political and corporate entities, as well as certain multilateral organizations, advocating for education to be congruent with the requirements of a globalized and digitized marketplace. ^1, 2^

From a critical and contextual pedagogical viewpoint, education constitutes a multifaceted social practice imbued with historical, ethical, and political significance, aimed at cultivating critical individuals and reinforcing the social fabric. ^3, 4^ Therefore, it is important to note that the educational function should not be reduced to a merely adaptive response to technological change. ^5^

The subordination of pedagogical objectives to technological means is one of the most persistent tensions in contemporary educational discourse. A narrative has emerged that prioritizes technology as the primary driver of educational transformation, while relegating pedagogical reflection to a secondary position, all in the name of efficiency, innovation, and competitiveness. ^6, 7^ In this context, teacher training is depicted as a continuous and expedited updating process, necessitated by technological urgency, rather than as a critical exercise associated with the essence of teaching. ^8^

Pedagogy should take a critical view of technology, according to. ^4, 9^ Instead of using technologies made in other countries, often by companies with goals other than education, the educational system should create technologies based on local knowledge and community needs. This point of view shows that the value of pedagogy is not in how well it works technically, but in building meaningful relationships with knowledge, encouraging critical thought, and taking care of the connections between people and communities. ^3^

The COVID-19 pandemic has illustrated that it is not the technological tools themselves that support educational processes; rather, it is the pedagogical knowledge, the contextualized use, and the ability of teachers to reorganize practices and adapt available tools in a creative, appropriate, and socially meaningful manner, based on the needs of their students. ^10^ Therefore, this demonstrates that it is not technology itself that transforms education, but rather its critical, contextual, and relational appropriation, ^11– 13^ which is accomplished in the classroom through the competencies that teachers develop. In this context, facing the widespread demotivation of 21st-century students, who no longer learn or engage in the same way as previous generations, ^14– 16^ teachers have resorted to various methodological strategies, among them gamification, to increase student motivation and engagement. ^17^

At the same time, education has undergone significant transformations through the integration of new technologies such as artificial intelligence (AI), which have contributed to the creation of more interactive learning environments. ^18^ Nonetheless, the incorporation of these emerging technologies requires situated, conscious pedagogical decisions, constructed in dialogue with the real needs of each educational community. ^10, 19, 20^

Moreover, this transformative potential must be critically examined, since educational innovation not only involves incorporating technologies such as generative artificial intelligence to create interactive environments like gamified platforms, but also articulating these tools with pedagogical approaches capable of responding to the challenges of inclusive, relevant, and ethical education. ^21^

The opinion article aims to analyze how the integration of artificial intelligence into deep gamification designs can contribute to authentic educational transformation, provided that such integration is guided by critical pedagogical principles and maintains a balance between the potential of technology and the formative power of teaching practices.

## Three emerging synergies between AI, gamification, and pedagogy for the development of 21st-century skills

The first synergy is concentrated on pedagogical knowledge and technological potential, the second is focused on gamification and AI as enablers of 21st-century skills, and the third is oriented toward these skills from the perspective of critical pedagogy. Three essential synergies can be determined.

The first synergy suggests that pedagogical knowledge and technological potential must be articulated: AI and gamification provide resources, but it is the teacher who ensures that they make sense in specific educational contexts. Training critical and competent citizens for the 21st century requires an ongoing dialogue between pedagogy and technology, where artificial intelligence and gamification are integrated from a critical approach that promotes meaningful, active, and contextualized learning. ^22^ From this perspective, educational innovation cannot be limited to the mere incorporation of devices or platforms but must be grounded in coherent pedagogical proposals that give purpose to their implementation. ^23^

To achieve real transformations, it is essential that artificial intelligence and gamification can be integrated into robust pedagogical frameworks, in which teachers assume a central role as designers and leaders of change-oriented processes. ^24^

From their professional autonomy, teachers promote educational innovation. ^25– 27^ Their task of validating, contextualizing, and enriching AI-generated content reaffirms their role as developers of critical thinking, mediators, and guides in teaching and learning processes, while integrating the emotional component that technology lacks. ^25, 28, 29^

Thus, the teacher’s role is crucial in designing and developing immersive and interactive experiences that are meaningful and foster sustained engagement among students. ^30^ This responsibility involves contextualizing the incorporation of technology, selecting and re-signifying resources according to students’ needs, pace, and realities, ensuring that innovations do not become ends in themselves but rather means to promote inclusive, critical, and culturally relevant learning. ^31– 33^

It is important to emphasize that this convergence requires teachers to critically analyze the scope and risks of AI implementation. Based on their knowledge and professional judgment, educator are the ones who lead pedagogical transformation, making decisions about the design, implementation, and continuous improvement of gamified environments.

The second synergy establishes that when gamification and artificial intelligence are combined, dynamic and motivating learning environments are generated, which foster the development of 21st-century skills. Also, the design of teaching environments can be enriched integrating AI and gamification, thereby fostering intrinsic motivation, increasing participation, improving academic performance, and promoting the inclusion of students with different learning styles. ^34^ The articulation between the adaptive power of AI and the motivational nature of gamification opens up new possibilities for transforming education into a meaningful, student-centered experience. ^32, 35^

According to, ^36^ this approach can contribute to the development of 21st-century skills such as creativity, communication, collaboration, and critical thinking, recognized by international organizations such as ISTE, ^37^ UNICEF, ^38^ OECD, ^39^ and UNESCO, ^40^ as well as other skills related to problem-solving, information management, technological appropriation, and innovation. ^41^

Likewise, by integrating playful elements into educational resources, gamification energizes teaching and learning through group activities and case resolution, which foster collaboration, communication, and critical thinking, ^42^ while also promoting digital learning and technological fluency in interactive, challenging, and contextualized scenarios. ^43^ In this regard, AI-powered gamified educational platforms can design adaptive and personalized experiences, ideal for developing critical thinking through problem-based learning and playful activities adjusted to students’ performance. ^44^

The third synergy considers that 21st-century skills and critical pedagogy give students with the opportunity to transform reality. In this sense, education should prepare students to become critical thinkers capable of adapting to new knowledge, solving problems, and actively participating in society. ^45^ This vision is linked to critical pedagogy, which emphasizes the importance of learners understanding their reality, making informed decisions, and proposing creative solutions to the challenges they face. Hence the need to promote the development of higher-order thinking skills in education, following the approach proposed by Freire, ^4^ so that students can analyze, evaluate, and apply knowledge in a conscious, critical, and constructive manner. ^46^

According to, ^47^ only a conscious appropriation in the educational sphere will allow us to transcend technical novelty and adopt a perspective inspired by the pedagogy of Freire, ^4^ in which 21st-century skills are not conceived merely as means to enhance employability, but as capacities oriented toward social transformation and full human development.

## AI in educational gamification: Between transformative potential and emerging risks

After acknowledging the relevance of the three synergies presented above, particularly that of gamification and artificial intelligence as a combination capable of boosting 21st-century skills, it is necessary to analyze in greater detail both the possibilities AI offers to enhance gamification and the risks associated with its implementation.

The integration of artificial intelligence into gamified designs emerges as a strategic reinforcement, since, as noted by Abbes et al., ^48^ its incorporation into education offers possibilities to create innovative teaching resources, transforms virtual instruction, and provides personalized and adaptive learning experiences that respond to the needs and pace of each student. From this perspective, and in complement to artificial intelligence, gamification plays a key role in the educational process due to its ability to increase motivation, strengthen commitment, and foster students’ autonomy. ^49, 50– 53^

Moreover, it aids in reinforcing learning by providing dynamic environments that facilitate both repetitive practice and the contextual application of knowledge. As emphasized by García-Martínez et al., ^54^ although gamification itself has numerous advantages in education, its efficacy is markedly enhanced when combined with the adaptive and personalized features afforded by artificial intelligence.

Nevertheless, it is important to stress that the incorporation of AI into gamified environments also entails risks. Hallifax et al. ^55^ point out that over-reliance on these systems could reduce teachers ‘influence and weaken personal interactions, which are essential for learning.’ Likewise, Liu ^49^ warns that an overload of feedback, interfaces, or complex tasks could overwhelm students, negatively affecting their motivation. Similarly, Hallifax et al. ^55^ emphasize that the implementation of adaptive gamification systems requires advanced infrastructure, accurate models of students’ behavior, and interdisciplinary collaboration, conditions that are not always easy to meet.

## From motivation to transformation: Deep gamification and AI in 21st-Century education

Gamification has demonstrated efficacy in education by enhancing student motivation. ^56^ It is not uniform, necessitating a distinction between two primary approaches: superficial gamification and deep gamification.

Although gamification, conceived as a methodological strategy ^57^ or as the incorporation of game elements into non-game contexts, ^58, 59^ has demonstrated its effectiveness in influencing human behavior, when limited to reward systems, its pedagogical potential is reduced, turning it into a practice centered solely on students’ superficial engagement. Therefore, it is essential that future teachers design gamified proposals that stimulate both intrinsic and extrinsic motivation and receive training that enables them to develop approaches capable of transcending external stimuli.

Based on this conceptual distinction, various studies such as ^32, 60– 63^ have identified key differences between shallow and deep gamification. While the former is limited to external reward mechanisms, such as points or leaderboards, the latter integrates immersive elements and pedagogical decisions aimed at generating meaningful experiences. ^61^ These differences encompass aspects such as pedagogical approach, the type of motivation, the impact on learning, design complexity, and the teacher’s role in the process. According to, ^32^ there are eight fundamental differences between shallow and deep gamification.

Firstly, regarding the teaching and learning process, del Olmo-Muñoz et al. ^64^ indicate that shallow gamification does not lead to substantial changes, whereas deep gamification introduces significant transformations in educational dynamics. Secondly, in terms of implementation difficulty, Mozelius ^61^ points out that shallow gamification is easy to apply, unlike deep gamification, which requires complex planning. Third, concerning pedagogical approaches, Hwang et al. ^63^ explain that shallow gamification is limited to structuring learning, whereas deep gamification directly intervenes in content. Fourth, with respect to the elements used, Mozelius ^61^ highlights that shallow gamification relies mainly on external rewards, in contrast with the meaningful and immersive game mechanics that characterize deep gamification. Fifth, Gurjanow et al. ^60^ underscore that shallow gamification requires technical skills such as programming and graphic design, whereas deep gamification demands expertise in game design with a pedagogical focus. Sixth, regarding motivational impact, the same authors state that shallow gamification produces limited effects, unlike the deep and lasting impact of deep gamification. Seventh, in terms of types of motivation, Mozelius ^61^ differentiates that shallow gamification fosters extrinsic motivation, whereas deep gamification promotes students’ intrinsic motivation.

Ultimately, regarding the duration of involvement, Söbke ^65^ suggests that superficial gamification is typically short-lived, but profound gamification persists over an extended period.

The differences between shallow and deep gamification reveal that gamification can offer different levels of depth, impact, and educational purpose. Accordingly, in order to achieve more elaborate gamified activities with sustainable effects over time, it is fundamental to integrate elements that foster students’ intrinsic motivation. ^66, 67^ In this regard, Turan et al. ^68^ highlight the importance of designing gamified experiences that not only use rewards but also promote meaningful transformations in educational processes.

In this context, recent research has explored gamification and identified both advantages and challenges associated with its implementation. For example, Dah et al. ^69^ warn that one of the main challenges of gamification is the prevalence of the “triad of badges, points, and leaderboards,” which fosters only extrinsic motivation and superficial engagement. While shallow gamification can spark interest through playful elements such as points and rewards, it does not significantly transform the learning experience. ^58, 70, 71^ Its effects are often ephemeral ^61, 72^ and tend to diminish in effectiveness when students lose interest in rewards, progressively reducing the effectiveness of these stimuli. ^73– 75^

Given these limitations, it is necessary to rethink gamification in the 21st century by incorporating elements that enhance intrinsic motivation and foster meaningful transformations. ^66– 68^ Some studies emphasize that deep gamification can generate radical changes in teaching–learning processes by integrating game mechanics into the core structure of the activity and creating narrative and immersive experiences that increase intrinsic motivation. ^72^

According to, ^41^ AI can strengthen the design of deep gamification experiences by generating personalized learning, providing immediate feedback, and creating more engaging interactive environments. From this perspective, artificial intelligence becomes a tool that allows teachers to redesign gamified experiences with greater pedagogical depth.

In this context, artificial intelligence constitutes a resource capable of reinforcing deep gamification by helping teachers design gamified environments in a dynamic, adaptive, and student-centered way, offering personalized learning, immediate feedback, and more attractive interactive experiences. ^41^ However, the implementation of this type of gamification faces challenges related to time, pedagogical planning, alignment with learning objectives, and scalability. ^60^

After establishing the relevance of integrating artificial intelligence into gamification designs, explaining how this combination fosters 21st-century skills, and highlighting the need for deep gamification experiences supported by AI, this opinion article argues that the integration of artificial intelligence into gamification processes can strengthen the design of deep gamification experiences by enabling highly personalized learning, adapted to students’ pace, styles, and needs, with immediate feedback and more engaging interactive environments.

The following parts provide the arguments and evidence that are in favor of the assertion that is made in this article.

## AI and deep gamification: A combination for personalized learning

After talking about how important it is to develop deep gamified settings, we need to look more closely at how the combination of deep gamification with artificial intelligence makes individualized experiences that fit each student’s needs, pace, and learning style.

Since educational systems must focus on the learner, personalizing earning is no longer simply a pedagogical goal but an urgent necessity. It is worth noting that, according to, ^48, 76^ artificial intelligence, when integrated into deep gamification designs, opens new possibilities for adequately adapting learning environments to the particularities of each student.

According to studies on deep gamification and the use of AI, this integration allows learning experiences to be personalized by adapting challenges, rewards, and feedback according to student performance, interests, and learning pace, thus fostering both interactive and motivating learning. ^44, 48, 49, 77^

Furthermore, according to, ^33, 78^ the synergy between AI and gamification allows the configuration of personalized educational experiences by analyzing student behavior in real time and dynamically adapting elements of the gamified environment, such as difficulty level, feedback, and rewards.

In contrast to some platforms that merely offer external rewards or standardized mechanics in shallow gamification designs, AI facilitates the creation of truly personalized experiences. Therefore, deep gamification enhanced by AI transcends playful interaction and becomes a powerful methodological strategy for personalizing learning in different educational contexts.

With respect to scaling and challenge adjustment, AI algorithms adjust task difficulty in real time, given that, according to, ^34, 49^ AI not only automates processes but also allows gamified elements (levels, challenges, rewards, and feedback) to be dynamically adapted based on students’ profiles and individual progress. In addition, according to, ^79^ generative artificial intelligence can accurately identify students’ misconceptions and use this information to generate appropriate feedback that addresses their learning needs.

Moreover, Markauskaite et al. ^80^ emphasize that AI becomes a valuable resource for teachers to optimize their time and focus on other important aspects of teaching, such as creating more complex and deeper learning opportunities. However, Giannakos et al. ^21^ stress the importance of teachers critically evaluating AI-powered feedback and complementing it with their own expertise.

This personalization capacity provided by AI aligns with the principles of deep gamification by facilitating immersive, meaningful learning experiences geared toward intrinsic motivation. According to, ^41^ through adaptive narratives and contextualized challenges, authentic and sustained student engagement can be promoted.

An example that illustrates the importance of personalization in the design of meaningful and culturally relevant linguistic experiences is the study by Xia et al. ^81^ In their research, the authors present the Intercultural Language Learning Intelligent System (CILS), which employs artificial intelligence to provide learning experiences tailored both to students’ profiles and to their cultural context. The results also highlight that adjusting teaching and learning strategies according to students’ cultural backgrounds and individual characteristics not only improves the effectiveness of the learning process but also fosters inclusive and meaningful communication in language learning environments.

Another example that demonstrates how the combination of artificial intelligence and gamification can personalize the level of difficulty, offer adaptive feedback, and track student performance in real time is the study by Laverde-Albarracín et al. ^82^ This research implemented a teaching strategy that integrated gamified resources and AI algorithms to generate automatic feedback and adapt challenges according to each student’s performance. AI tools were also used to monitor response time and accuracy in solving exercises, enabling personalized adjustments. Notably, results revealed a 40% increase in the resolution of complex problems in the experimental group compared to the control group.

Similarly, the study by Mohammed and Jesudas ^83^ shows how the integration of AI and gamification transforms language learning. AI offers personalized learning paths, immediate feedback, and content adapted to individual needs, while gamification fosters engagement and motivation through dynamic experiences. This study concludes that the combination of AI and gamification promotes an interactive learning environment that strengthens students’ language skills, sustains motivation and engagement over time, reinforces learning through gamified repetition, generates immersive, contextualized, and culturally relevant experiences, encourages collaboration and competition, and facilitates autonomous learning.

Furthermore, Liu, ^49^ Cabrera Félix and Román Santana ^84^ highlight that incorporating artificial intelligence into gamified environments allows to implement personalized pedagogical approaches tailored to students’ individual needs, while also fostering interaction and generating meaningful educational experiences.

Moreover, Bachiri et al., ^35^ Kassenkhan et al., ^44^ Pardim et al., ^78^ Martínez et al., ^85^ Pelletier et al. ^86^ demonstrate that AI-enhanced gamified designs not only increase students ’participation but also improve feedback and adapt activities based on students’ performance and individual characteristics. These studies reinforce the idea that integrating AI into gamified design is an effective strategy to personalize learning, stimulate sustained progress, and address classroom diversity.

Likewise, Abbes et al. ^48^ argue that incorporating artificial intelligence into gamified environments designed by teachers offers an opportunity to transform how students learn, as it makes it possible to create unique learning paths for each student, responding to their individual needs and stimulating continuous learning progress.

In this context, it is essential to consider students’ diverse learning styles (visual, verbal, active, reflective, and sensory) when designing AI-supported gamified environments. ^87^ Adequate alignment between challenges and learning objectives with students’ interests and styles fosters active participation and stimulates problem-solving skills, allowing each student to progress at their own pace. ^88, 89^ Thus, teachers’ identification of these learning styles enables AI to contribute to the creation of personalized resources and the consolidation of deep gamification experiences. ^90^

In addition, artificial intelligence, by efficiently filtering and organizing large volumes of information, facilitates teachers’ ability to make relevant pedagogical adjustments, adapting both teaching methods and resources to students’ individual characteristics. ^91^ Platforms such as *Classcraft*, *MathCityMap*, and *@MyClassGame* provide concrete examples, as they allow for the implementation of both shallow gamification strategies, based on points, badges, and leaderboards, and deep gamification strategies focused on narratives, meaningful missions, and collaborative dynamics. In this sense, it is up to teachers, based on their formative objectives, to determine which approach is the most relevant. ^54^

Although research evidence supports the potential of AI in gamification designs to improve personalization of learning, it is important to stress that, according to, ^62^ pedagogical creativity and decision-making remain the responsibility of the teacher, since the teacher plays an important role in the personalization process and trains the algorithms to obtain better results.

Finally, it’s important to talk about the problems that come with customisation. The research conducted by Gao et al. ^73^ concluded that the customization algorithms in the AI-enhanced gamified platform ShouTi Fitness require refinement to ensure that recommendations are tailored to students’ proficiency levels, as 10% of participants said that certain suggestions were redundant. Moreover, Hallifax et al. ^55^ caution that modifying game aspects to correspond with students’ psychological profiles presents an ethical quandary: personalized gamification may exploit learners and curtail their agency. In the same way, too much adaptation might make pupils only interact with things they are already comfortable with, which can slow their growth and make it harder for them to deal with new problems.

## AI and deep gamification: An innovative alliance with real impact on learning

As noted in the previous section, the application of AI in education not only personalizes learning within gamified designs but also opens new possibilities for the development of teaching resources, the creation of interactive environments, and the comprehensive transformation of teaching and learning processes. ^48^

In this regard, several studies have shown that the integration of AI improves the efficiency and adaptability of teaching activities, while enabling dynamic, meaningful, and student-centered learning experiences. ^34, 63^ These findings reinforce the idea that when AI is articulated with methodological strategies such as deep gamification, it not only facilitates the personalization of learning but also generates a real and sustainable impact on educational outcomes.

For example, Bennani et al. ^34^ highlight that incorporating gamified challenges and interactive components into immersive educational environments optimizes academic performance, fosters motivation, and stimulates creativity. They also propose integrating artificial intelligence to facilitate students’ access to information, taking into account individual characteristics and profiles.

Similarly, Erbaşı et al. ^76^ argue that the convergence of AI and gamification produces a radical change, as it enables the design of efficient and meaningful learning experiences. Essentially, this union represents an effective set of tools to develop engaging learning systems with better academic results.

Studies on the combination of AI and gamification, such as, ^36, 41^ agree that integrating artificial intelligence into deep gamified environments constitutes an innovative strategy to transform learning. The following section addresses why this innovative alliance should be seriously considered.

Recent empirical evidence expands this perspective. Gao et al. ^73^ note that the synergy between deep gamification and artificial intelligence increases students ‘motivation and engagement by creating interactive environments in which learners are actively involved in their learning process. Gamified designs stimulate exploration, decision-making, and problem-solving in playful contexts, while AI enriches these experiences with personalization, adaptive feedback, and real-time analysis. In this way, meaningful learning and autonomy are strengthened, and academic performance is improved. ^49, 62, 92^

The study by Liu ^49^ provides complementary evidence. With a sample of 486 university students from an English teaching program in China, it compared three AI-powered gamification strategies: adaptive learning paths, conversational agents, and interactive storytelling. The results showed that the combination of gamification and AI increased motivation, personalized the learning experience, and enriched learning, particularly through interactive storytelling that incorporated diverse cultural contexts.

Specifically, students valued the immediate feedback from conversational agents, the cultural richness of interactive storytelling, and the personalization of adaptive learning paths. Scoreboards, unlockable content, and individualized challenges sustained interest and reinforced intrinsic motivation, which translated into significant improvements in both language proficiency and student engagement. In contrast, the control group, which followed a conventional course without AI or gamification, showed no significant progress, confirming that the deep integration of AI and gamification transforms the learning experience by making it more meaningful, motivating, and personalized.

Although the study by Liu ^49^ is not explicitly framed within a critical pedagogy perspective, the inclusion of cultural elements into interactive storytelling can be interpreted as an attempt to link learning to meaningful contexts for students. This aspect aligns, at least partially, with the arguments of Teräs, ^6^ Williamson, ^7^ Facer, ^8^ who emphasize the importance of addressing the realities and needs of local communities.

On the other hand, artificial intelligence facilitates teachers’ work, since in deep gamified environments AI allows real-time analysis of students’ behavior and progress, automatic adaptation of levels and rewards, and identification of learning styles and emotional states. These capabilities, according to, ^91, 93, 94^ enable teachers to focus on the pedagogical dimension, intervene in a timely manner, and address students’ needs in a personalized way.

In summary, the combination of deep gamification and artificial intelligence entails significant implications for teaching practice and the optimization of teaching and learning processes at all educational levels. In this sense, teachers should rely on AI to design gamified environments that respond to students’ interests and needs. ^50^ This requires moving toward deeper pedagogical proposals that integrate meaningful narratives, adaptive feedback, and contextualized challenges, overcoming mechanical models focused exclusively on extrinsic rewards. ^95, 96^

Similarly, it is imperative that students engage actively in their educational journey, fostering the growth of autonomy and decision-making abilities. Strategies like creating personalized avatars or customizing aspects of the gamified environment can enhance identification, boost engagement, and reinforce commitment to learning, so promoting intrinsic motivation.

The utilization of gamification analytics technologies, such as GamAnalytics, in AI-enhanced gamified environments enables educators to more precisely track student interactions with gamified systems. By visualizing and analyzing learning data produced by AI, such as speech, gestures, and student action logs, educators receive feedback to modify game dynamics and enhance gamification designs, resulting in increased engagement, motivation, and learning outcomes. ^21, 97^ This establishes a co-creation process between educators and artificial intelligence, designed to enhance the educational experience and revolutionize teaching and learning methodologies.

## Between potential and the gap: Ethical and technological challenges of AI-optimized Gamification

Despite the immense pedagogical potential of the combination of artificial intelligence and profound gamification, its implementation presents substantial challenges that must not be disregarded. The following are particularly noteworthy among these:

The first challenge concerns data protection, ethics, and privacy. Gamification mediated by artificial intelligence collects large volumes of information about students, including their academic progress, in-game behavior, preferences, and even demographic data, which poses risks if the security and confidentiality of such records are not guaranteed. ^98, 99^ To address this issue, it is essential to establish strong security measures such as data protection protocols and audits of AI models to detect bias and prevent misuse. ^35, 41^

The second challenge has to do with avoiding excessive dependence on technology. AI should be understood as a means and not an end in educational processes. ^91^ Therefore, it is not advisable to fully delegate the design of gamified experiences to artificial intelligence, especially regarding content personalization, the difficulty of challenges and narratives, since game mechanics must always respond to pedagogical objectives. ^41, 100^ In this sense, AI should play a complementary role, contributing to analysis, monitoring, and optimization, while pedagogical design remains the responsibility of the teacher. ^101^

The third challenge relates to the technological and infrastructural gap. Significant inequalities persist in access to, use of, and proficiency with information, communication, and artificial intelligence technologies. ^63^ UNESCO, ^40^ in its 2023 agenda, stresses that young people must be guaranteed access to both formal and informal educational experiences that broadly integrate technology to ensure greater equity in opportunities.

This gap affects both teachers and students who lack technological resources in their educational institutions. ^83, 85^ Neubaum et al. ^102^ showed that during the pandemic, advances in digital skills mainly benefited young people with greater resources, thus deepening inequalities rather than reducing them. According to, ^91^ this exclusion stems from social, demographic, and educational factors that limit equitable access to the benefits of technological advances.

With respect to infrastructure, the need to provide educational institutions with both physical and digital resources has become evident: devices, connectivity, educational software, digital platforms, and tools for developing AI-supported gamified experiences. ^85^ The lack of such resources restricts the effective implementation of new proposals, especially in developing countries where investment in technological equipment remains insufficient. ^91^

The fourth challenge involves the cultural and pedagogical appropriation of technology. As Feenberg ^103^ argues, technologies are not neutral, but neither are they closed: they can be adapted and reconfigured according to context. However, as Watters ^104^ warns, the problem does not lie in the rigidity of technology but in the uncritical adoption of external models, particularly from the Global North, without adapting them to local contexts, languages, and teaching practices. De Sousa Santos and Meneses ^105^ reinforce this idea, by pointing out that it is not about incorporating tools without reflection but about questioning their meaning within situated educational processes. Instead of replicating foreign uses, it is more relevant to start from our own educational and cultural needs. Innovation, in this sense, should be conceived as situated creation rather than mere imitation.

The fifth challenge relates to teacher training, a fundamental aspect for harnessing the potential of AI-mediated deep gamification. According to, ^48^ the lack of specialized preparation significantly limits its impact in education. Dah et al. ^69^ agree that the effectiveness of gamification depends on multiple factors: design quality, ^106^ theoretical grounding, ^107, 108^ standardization of elements, ^107^ individual differences, ^109^ and contextual particularities. ^110^ These conditions underscore the need for teachers trained in gamification software, ^111^ capable of adapting resources to their educational contexts and to the characteristics of their students. ^112^

In order to progress toward meaningful implementation, it is imperative to provide training to both in-service and preservice teachers on the development of gamified experiences that incorporate AI as a support tool. This training should encompass the acquisition of critical digital literacy skills, the comprehension of adaptive systems, and the mastery of resources such as immersive narratives, dynamic feedback, and contextualized challenges.

Finally, despite the challenges, the future of AI-supported gamification is promising. This technological convergence can transform learning into a more dynamic and motivating experience by encouraging student participation and allowing the continuous adaptation of gamification designs throughout the learning process. ^97^ In this context, addressing current challenges from a critical and proactive perspective will make it possible to fully leverage the transformative potential of AI in deep gamification environments. Ultimately, true innovation, as Örpek et al. ^33^ argue, will depend on whether educational institutions and educators manage to balance the intelligent use of technology with situated pedagogical objectives, ensuring that educational practices respond to the needs of local communities.

## Conclusions

This opinion piece critically examines how the use of artificial intelligence might improve deep gamification ideas. This synergy goes beyond approaches based on external rewards and opens the door to a pedagogical transformation centered on the creation of authentic, adaptive, and motivating experiences that foster meaningful learning, stimulate intrinsic motivation, and strengthen students’ commitment to their own learning process.

The analysis underscores the significance of acknowledging that artificial intelligence does not supplant the teacher’s function but rather augments it by providing resources for personalization, real-time feedback, and automated material development. The gamification of educational experiences relies on the educator, who, from a critical standpoint, crafts narratives and challenges while considering the reality, interests, needs, and learning styles of their pupils.

The reflection also makes it clear that combining AI and deep gamification is not only a technological upgrade; it is a change in the way we talk about human and artificial intelligence. This change means that teachers need to create meaningful learning experiences instead of just giving students fun activities to do to keep them interested in class. ^113^ They also need to update their teaching methods to help students learn 21st-century skills like creativity, collaboration, communication, and critical thinking, while also dealing with ethical issues like privacy, technological equity, and cultural relevance.

Ultimately, it is crucial to perceive artificial intelligence as a pivotal instrument for converting static gamification into dynamic, adaptable, and learner-centered educational experiences. ^34^ The revolutionary potential will be actualized alone via deliberate planning, robust teacher training, and the ethical and contextual application of technology. Thus, profound gamification enhanced by artificial intelligence would facilitate authentic educational transformations in the contemporary period.

## F1000 AI policy

We have read and agree to comply with the F1000Research AI Policy. We confirm that, in accordance with this policy, ChatGPT-5 was used for style correction review. The use of generative AI was carried out under the supervision of the authors, with full transparency and rigorous review.

## Data Availability

No data are associated with this article.
