# Deep Gamification and Artificial Intelligence as Catalysts of Educational Transformation

**DOI:** 10.12688/f1000research.171453.2

**Published:** 2025-12-22

**Authors:** Diana Milena Patiño Barriga, Ana Dolores Vargas Sánchez, Paloma Valdivia Vizarreta

**Affiliations:** 1 Universidad de La Sabana, Chia, Cundinamarca, Colombia; 2 Universitat Autonoma de Barcelona, Barcelona, Cataluña, Spain

**Keywords:** Gamification, artificial intelligence, learning, innovation, social transformation

## Abstract

This opinion article examines the convergence between artificial intelligence (AI) and gamification in learning environments, with an emphasis on deep gamification designs aimed at creating interactive and meaningful experiences that transcend extrinsic motivation. The introduction sets the context by presenting AI as a tool that must be critically analyzed within the framework of critical pedagogy, which underscores the importance of adopting technology reflectively, placing the student at the center, and responding to the real needs of the community. The body of the article develops this claim: the integration of artificial intelligence into deep gamification designs can contribute to genuine educational transformation, provided that such integration is guided by critical pedagogical principles and maintains a balance between the potential of technology and the formative power of teaching practices. Arguments related to this claim are provided, including the use of AI in gamified designs to create personalized learning experiences based on students’ needs, rhythms, and learning styles; to transform the way students learn; to offer new educational resources; to adapt elements such as difficulty levels, challenges, and feedback in real time; and to develop engaging learning systems that lead to better academic outcomes. The conclusion emphasizes the significance of instructors utilizing critical pedagogy to direct AI-optimized gamification designs by incorporating culture, creativity, and critical awareness as pillars of educational transformation. This approach enables the surmounting of the obstacles presented by this synergy.

## Introduction

In the 21st century, the integration of digital technologies into modern education has been characterized by narratives advanced by political and corporate entities, as well as certain multilateral organizations, advocating for education to be congruent with the requirements of a globalized and digitized marketplace. ^1, 2^

From a critical and contextual pedagogical viewpoint, education constitutes a multifaceted social practice imbued with historical, ethical, and political significance, aimed at cultivating critical individuals and reinforcing the social fabric. ^3, 4^ Therefore, it is important to note that the educational function should not be reduced to a merely adaptive response to technological change. ^5^

The subordination of pedagogical objectives to technological means is one of the most persistent tensions in contemporary educational discourse. A narrative has emerged that prioritizes technology as the primary driver of educational transformation, while relegating pedagogical reflection to a secondary position, all in the name of efficiency, innovation, and competitiveness. ^6, 7^ In this context, teacher training is depicted as a continuous and expedited updating process, necessitated by technological urgency, rather than as a critical exercise associated with the essence of teaching. ^8^

Pedagogy should take a critical view of technology, according to ^4, 9^ instead of using technologies made in other countries, often by companies with goals other than education, the educational system should create technologies based on local knowledge and community needs. This point of view shows that the value of pedagogy is not in how well it works technically, but in building meaningful relationships with knowledge, encouraging critical thought, and taking care of the connections between people and communities. ^3^

The COVID-19 pandemic has illustrated that it is not the technological tools themselves that support educational processes; rather, it is the pedagogical knowledge, the contextualized use, and the ability of teachers to reorganize practices and adapt available tools in a creative, appropriate, and socially meaningful manner, based on the needs of their students. ^10^ Therefore, this demonstrates that it is not technology itself that transforms education, but rather its critical, contextual, and relational appropriation, ^11– 13^ which is accomplished in the classroom through the competencies that teachers develop. In this context, facing the widespread demotivation of 21st-century students, who no longer learn or engage in the same way as previous generations, ^14– 16^ teachers have resorted to various methodological strategies, among them gamification, to increase student motivation and engagement. ^17^

At the same time, education has undergone significant transformations through the integration of new technologies such as artificial intelligence (AI), which have contributed to the creation of more interactive learning environments. ^18^ Nonetheless, the incorporation of these emerging technologies requires situated, conscious pedagogical decisions, constructed in dialogue with the real needs of each educational community. ^10, 19, 20^

Moreover, this transformative potential must be critically examined, since educational innovation not only involves incorporating technologies such as generative artificial intelligence to create interactive environments like gamified platforms, but also articulating these tools with pedagogical approaches capable of responding to the challenges of inclusive, relevant, and ethical education. ^21^

The opinion article aims to analyze how the integration of artificial intelligence into deep gamification designs can contribute to authentic educational transformation, provided that such integration is guided by critical pedagogical principles and maintains a balance between the potential of technology and the formative power of teaching practices.

## Method

This opinion article is grounded in a thematic conceptual synthesis supported by a conceptual review and a critical synthesis of the literature on artificial intelligence, gamification and critical pedagogy. The purpose of the analysis was to examine and compare key scholarly contributions, and to identify conceptual patterns, convergences, and tensions across recent and relevant sources drawn from studies addressing the integration of deep gamification and AI in recognized databases such as Scopus and Web of Science.

The analytical process followed an iterative and interpretive procedure. First, relevant publications were selected based on their conceptual contribution to understanding deep and shallow gamification, AI-supported learning environments and the development of 21st-century skills. Second, these texts were read closely to extract significant ideas, pedagogical arguments, and recurring constructs. Our review revealed that, despite the growing importance of artificial intelligence and gamification in education, few studies explore their intersection, particularly within the context of deep gamification. Third, units of meaning were coded and clustered, allowing higher-order conceptual themes to emerge. This process resulted in six major categories that structure the argument developed in this article: deep and shallow gamification as a framework to understand AI integration, synergies between gamification and pedagogy, benefits of integrating AI into deep gamified designs, risks of incorporating AI into gamified designs, the role of teachers within AI-optimized gamification, and ethical and technological challenges (see appendix).

Throughout the article, we include examples from current research to illustrate how AI supports deep gamification through personalization, adaptative challenges, real-time feedback, and immersive narratives. This method privileges conceptual clarity and integrative synthesis over exhaustive coverage, aligning with the reflective purpose of this opinion article.

## Three emerging synergies between AI, gamification, and pedagogy for the development of 21st-century skills

**Figure 1.**
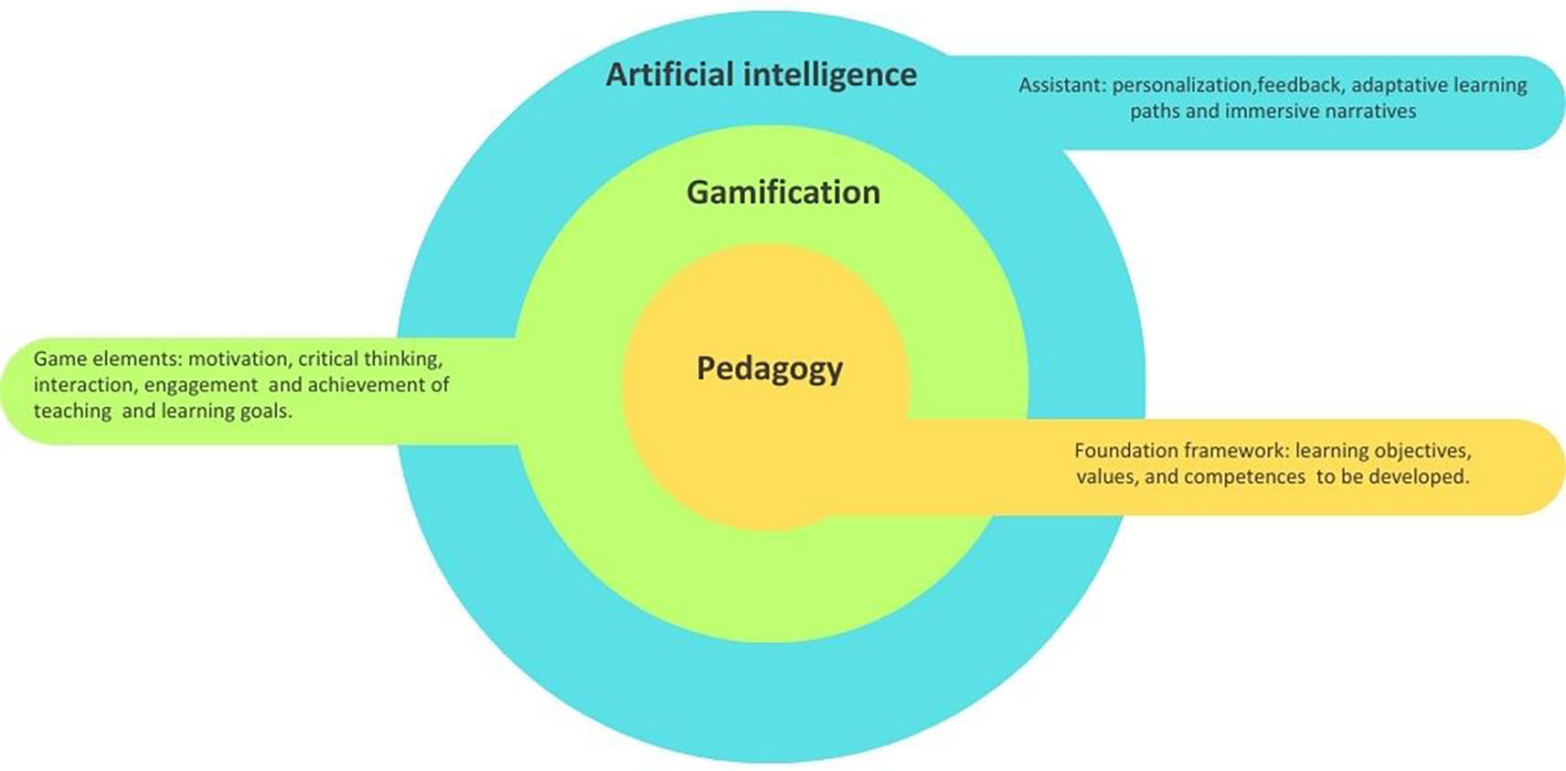
Relationship between pedagogy, gamification and artificial intelligence. Note: The figure illustrates a three-layer model in which pedagogy forms the core foundation of teaching and learning; gamification emerges as an intermediate layer integrating game elements to transform traditional learning environments and artificial intelligence functions as an outer assistant layer enabling personalization, adaptative pathways, and immersive narrative design. Own source elaborated, adapting the multi directional model of pedagogical reasoning for gamification ^23^ and the AI oriented pedagogical model. ^22^

The relationship between pedagogy, gamification and artificial intelligence is fundamentally interdependent. Rather than functioning as separate components, they interact to shape how students engage, participate, and learn in contemporary learning environments (see Figure 1). In the three-layer model, pedagogy occupies the central position because it provides the foundational core that defines learning objectives, values, and competencies. It involves the decisions teachers make about how students learn most effectively within specific contexts and emerges from the relational interactions between teachers, students and the learning objects. ^22^

Gamification is positioned in the intermediate layer because it depends on pedagogical reasoning for its meaningful implementation. When grounded in sound pedagogical design, gamification can transform traditional learning environments by fostering motivation, facilitating critical thinking and interaction, engaging students, and supporting the achievement of specific teaching and learning goals. ^23^

Finally, artificial intelligence functions as an external, supportive layer that enhances learning experiences through personalization, immediate feedback, adaptive learning paths, and immersive narratives. Instead of replacing teachers, AI collaborates with them in the co-construction of learning pathways, shifting from a hierarchical, top-down approach to a more dynamic and relational pedagogy. This creates a more collaborative relationship in which educators, students, and AI technologies participate, transforming the educational experience. ^22^

Altogether, the three-layer model highlights that AI and gamification only become pedagogically meaningful when guided by intentional, coherent teaching practices. This conceptual foundation enables a deeper understanding of how they interact in real learning contexts. Taking this into account, this section outlines three key synergies through which AI-enhanced gamification can support meaningful educational practices.

Synergy 1: Articulating pedagogical knowledge and pedagogical potential

The first synergy suggests that pedagogical knowledge and technological potential must be articulated: AI and gamification provide resources, but it is the teacher who ensures that they make sense in specific educational contexts. Training requires an ongoing dialogue between pedagogy and technology, where artificial intelligence and gamification are integrated from a critical approach that promotes meaningful, active, and contextualized learning. ^24^ From this perspective, educational innovation cannot be limited to the mere incorporation of devices or platforms but must be grounded in coherent pedagogical proposals that give purpose to their implementation. ^25^

To achieve real transformations, it is essential that artificial intelligence and gamification can be integrated into robust pedagogical frameworks, in which teachers assume a central role as designers and leaders of change-oriented processes. ^26^

From their professional autonomy, teachers promote educational innovation. ^27– 29^ Their task of validating, contextualizing, and enriching AI-generated content reaffirms their role as facilitators and guides in teaching and learning processes, while integrating the emotional component that technology lacks. ^27, 30, 31^

This autonomy should be understood not only as the ability to make important decisions based on professional expertise, but also as the capacity to adapt AI-generated content and supervise the behavior of algorithmic systems and other emerging technologies in education. It involves critically reviewing algorithmic outputs, identifying potential biases, and ensuring that personalization and adaptive feedback remain aligned with students’ needs and cultural contexts. Moreover, it is essential to recognize teachers’ agency in decision-making regarding AI integration, involving them in the design and implementation of institutional and policy frameworks and enabling them to adapt emerging technologies in active and creative ways. ^32^

Thus, the teacher’s role is crucial in designing and developing immersive and interactive experiences that are meaningful and foster sustained engagement among students. ^33^ This responsibility involves contextualizing the incorporation of technology, selecting and re-signifying resources according to students’ needs, pace, and realities, ensuring that innovations do not become ends in themselves but rather means to promote inclusive, critical, and culturally relevant learning. ^34– 36^

It is important to emphasize that this convergence requires teachers to critically analyze the scope and risks of AI implementation. Based on their knowledge and professional judgment, educator are the ones who lead pedagogical transformation, making decisions about the design, implementation, and continuous improvement of gamified environments.

Synergy 2: AI and gamification as enablers of 21st century skills

The second synergy establishes that when gamification and artificial intelligence are combined, dynamic and motivating learning environments are generated, which foster the development of 21st-century skills. Also, the design of teaching environments can be enriched integrating AI and gamification, thereby fostering intrinsic motivation, increasing participation, improving academic performance, and promoting the inclusion of students with different learning styles. ^37^ The articulation between the adaptive power of AI and the motivational nature of gamification opens up new possibilities for transforming education into a meaningful, student-centered experience. ^35, 38^

According to, ^39^ this approach can contribute to the development of 21st-century skills such as problem solving, information management, technological appropiation and innovation, ^40^ as well as creativity, communication, collaboration, and critical thinking, recognized by international organizations such as ISTE, ^41^ UNICEF ^42^ OECD, ^43^ and UNESCO. ^44^

Likewise, by integrating playful elements into educational resources, gamification energizes teaching and learning through group activities and case resolution, which foster the 21st century skills mentioned above, ^45^ while also promoting digital learning and technological fluency in interactive, challenging, and contextualized scenarios. ^46^ In this regard, AI-powered gamified educational platforms can design adaptive and personalized experiences, ideal for developing critical thinking through problem-based learning and playful activities adjusted to students’ performance. ^47^

Synergy 3 From skill acquisition to social transformation through critical pedagogy

The third synergy proposes moving beyond the development of skills for individual success to offer students opportunities to transform reality from the perspective of critical pedagogy. In this sense, education should prepare students to become critical thinkers capable of adapting to new knowledge, solving problems, and actively participating in society. ^48^ This vision aligns with critical pedagogy, which emphasizes the importance of students interpreting their contexts, making informed decisions, and generating creative solutions to the challenges they face. Following the approach proposed by Freire 4, this implies fostering tstudents’ capacity to analyze, evaluate, and apply knowledge in a conscious, critical, and constructive ways. ^49^

According to, ^50^ only a conscious appropriation of emerging technologies within educational practices allows us to transcend technical novelty and adopt a perspective inspired by Freire’s pedagogy. ^4^

This synergy highlights that the integration of gamification and AI must be grounded in a critical pedagogical stance that empowers students to question, participate, and co-construct knowledge. In a deeply gamified environment enriched with AI, gamified experiences inspired by popular narratives such as *Game of Thrones* can be transformed into powerful opportunities for critical inquiry.

## AI in educational gamification: Between transformative potential and emerging risks

After acknowledging the relevance of the three synergies presented above, particularly that of gamification and artificial intelligence as a combination capable of boosting 21st-century skills, it is necessary to analyze in greater detail both the possibilities AI offers to enhance gamification, the risks associated with its implementation and the competences teachers need to develop to face algorithmic bias and ethical oversight.

The integration of artificial intelligence into gamified designs emerges as a strategic reinforcement, since, as noted by Abbes et al., ^51^ its incorporation into education offers possibilities to create innovative teaching resources, transforms virtual instruction, and provides personalized and adaptive learning experiences that respond to the needs and pace of each student. From this perspective, and in complement to artificial intelligence, gamification plays a key role in the educational process due to its ability to increase motivation, strengthen commitment, and foster students’ autonomy. ^52– 56^

Moreover, it aids in reinforcing learning by providing dynamic environments that facilitate both repetitive practice and the contextual application of knowledge. As emphasized by García-Martínez et al., ^57^ although gamification itself has numerous advantages in education, its efficacy is markedly enhanced when combined with the adaptive and personalized features afforded by artificial intelligence.

Nevertheless, it is important to stress that the incorporation of AI into gamified environments also entails risks. Hallifax et al. ^58^ point out that over-reliance on these systems could reduce teachers ‘influence and weaken personal interactions, which are essential for learning.’ Likewise, Liu ^52^ warns that an overload of feedback, interfaces, or complex tasks could overwhelm students, negatively affecting their motivation. Similarly, Hallifax et al. ^58^ emphasize that the implementation of adaptive gamification systems requires advanced infrastructure, accurate models of students’ behavior, and interdisciplinary collaboration, conditions that are not always easy to meet.

The incorporation of artificial intelligence into gamified educational settings necessitates a focus on educator autonomy and ethical supervision, especially as adaptive systems progressively influence classroom dynamics. According to ^59^ AI Competency Framework for Teachers, AI distinguishes itself from earlier iterations of educational technologies by its ability to replicate aspects of human judgment, prediction, and decision-making. This distinctive attribute poses potential risks to human agency, particularly when educators develop excessive reliance on automated recommendations or adaptive learning routes. For AI-enhanced gamification to maintain a pedagogically sound foundation, educators must therefore exercise ongoing interpretive authority, making critical decisions regarding when, how, and to what extent AI-generated feedback or adaptive challenges are consistent with learning objectives and students’ requirements.

A fundamental aspect of instructor autonomy within AI-enhanced environments is the ability to mitigate algorithmic bias. There are various forms of bias in education, such as selection bias, which arises when data do not accurately represent the entire student population; confirmation bias, which involves the tendency to seek information that reinforces existing beliefs; stereotypical bias, which encompasses assumptions based on gender stereotypes; and cultural bias, which manifests through language or examples that are irrelevant to specific cultural contexts. Concerning algorithmic biases, UNESCO ^59^ highlights that exclusion and discrimination may be inherently embedded within AI models and datasets, leading to automated decisions that perpetuate inequalities across gender, language, and socioeconomic status.

Within gamified designs, where performance data influence difficulty levels, reward systems, classifications, and feedback mechanisms, educators must rigorously oversee AI outputs to detect instances of inequitable treatment. This entails evaluating the pedagogical legitimacy of adaptive suggestions, situating automated recommendations within students’ sociocultural contexts, and intervening when gamified feedback inadvertently disadvantages specific groups of students. In this context, educators ensure that AI-enhanced gamification promotes inclusion, diversity, and equitable engagement. ^44^

In relation to ethical oversight, teachers have to rely on robust conceptual frameworks that allow them to make informed critical decisions about the validity, fairness and contextual relevance of algorithmic outputs. ^32^ Through this reflective stance, they act as ethical mediators who detect and mitigate algorithmic bias ensuring that AI-optimized gamification experiences align with pedagogical goals and cultural relevant learning narratives, thus preventing automated systems from losing sight of the educational purpose.

In relation to ethical oversight, teachers must rely on robust conceptual frameworks that enable them to make informed, critical decisions about the validity, fairness, and contextual relevance of algorithmic outputs. ^32^ Through this reflective stance, they act as ethical mediators who detect and mitigate algorithmic bias, ensuring that AI-optimized gamification experiences align with pedagogical goals and culturally relevant learning narratives, thus preventing automated systems from losing sight of the educational purpose.

Teachers mediate algorithmic bias by critically evaluating algorithmic outputs. For instance, they apply algorithmic awareness, as a key component of digital literacy, to analyze AI feedback; they ensure equity in learning opportunities by avoiding exclusive reliance on AI-driven ability grouping and by providing support for students who face difficulties; they monitor learning processes in gamified experiences by checking dashboards to identify potential biases in real-time interactions; and they intervene directly in the classroom by deciding whether to keep or modify AI recommendations and by adjusting these suggestions. Moreover, as suggested by, ^60^ it is important to require bias audits and to create communities of practice that allow teachers to share effective strategies for addressing this challenge.

To effectively fulfill the role of ethical mediator and make informed, critical decisions, teachers need to develop specific competencies. According to, ^59^ fifteen competencies required for effective ethical oversight are organized into five dimensions: a human-centred mindset, ethics of AI, AI foundations and applications, AI pedagogy, and AI for professional learning, which are articulated within a three-level progression model. It is important to note that these five competency areas are integrated across every progression level.

The first progression level, *Acquire*, outlines the essential AI literacy that all teachers must develop in order to evaluate, select, and use AI tools appropriately in their teaching practice. This foundational stage includes understanding how AI models are trained, recognizing potential benefits and risks, and identifying ethically relevant issues such as human rights, data privacy, and human-centred design. According to the UNESCO *AI Competency Framework for Teachers*, ^59^ this level comprises five core competencies. The first, human agency, emphasizes that teachers must understand that AI systems are human-created and human-guided, and that the decisions embedded in their design can significantly influence learners’ autonomy and rights. The second, ethical principles, requires teachers to grasp the basic ethical issues surrounding AI and the principles that underpin responsible human–AI interactions. The third, basic AI techniques and applications, involves acquiring foundational conceptual knowledge—what AI is, how models are trained, and how to determine whether particular AI tools are pedagogically appropriate and properly validated. The fourth competency, AI-assisted teaching, expects teachers to identify the pedagogical benefits of AI tools for enhancing lesson planning, instruction, and assessment, while actively mitigating associated risks. Finally, enabling lifelong professional learning highlights the importance of teachers using AI tools to support their own professional growth, reflective practice, and ongoing adaptation to evolving educational demands. ^59^

The initial progression level, Acquire, delineates the fundamental AI literacy that all educators are required to cultivate to effectively assess, choose, and utilize AI tools within their instructional practices. This level encompasses a comprehension of the training processes of AI models, an assessment of their potential benefits and associated risks, and the identification of ethically significant issues such as human rights, data privacy, and human-centered design. According to the UNESCO AI Competency Framework for Teachers, ^59^ this level encompasses five fundamental competencies. The initial principle, human agency, underscores the importance for educators to recognize that AI systems are developed and directed by humans, and that the decisions incorporated into their design can substantially impact students’ autonomy and rights. The second, ethical principles, mandates that educators understand the fundamental ethical considerations related to AI and the principles that underpin responsible interactions between humans and AI. The third, fundamental category of AI techniques and applications, encompasses the acquisition of essential conceptual knowledge, including a comprehension of what artificial intelligence entails, the processes involved in training models, and the criteria for assessing the pedagogical suitability and validation of specific AI tools. The fourth competency, AI-assisted teaching, requires educators to recognize the pedagogical advantages of AI tools in augmenting lesson planning, instruction, and assessment, while proactively addressing and mitigating related risks. The fifth, which facilitates continuous professional development, emphasizes the significance of educators employing AI tools to enhance their ongoing professional growth and reflective practices. ^59^

The second level, Deepen, outlines the intermediate competencies necessary for teachers to develop meaningful pedagogical strategies that ethically and purposefully integrate AI. Teachers at this level must exhibit human accountability in the integration of AI into their lessons, critically evaluate AI tools for ethical implications, ensure safe and responsible usage, promote equity, inclusion, and diversity, comply with legal and institutional frameworks, and apply ethical principles in the identification, selection, evaluation, and implementation of AI tools to improve their teaching practices. ^59^ This level includes five competencies. The first aspect, human accountability, underscores the necessity for teachers to exhibit a comprehensive understanding of human responsibility regarding the appropriate use and implementation of AI. The second principle, safe and responsible use, emphasizes the necessity for educators to internalize ethical guidelines for the responsible and safe application of AI. This includes respecting data privacy and intellectual property rights, which should be applied when evaluating, utilizing, and creating content with AI tools. The third competency, application skills, emphasizes that educators must proficiently utilize AI tools sanctioned for educational use, while systematically integrating ethical principles into their practice. The fourth aspect, AI–pedagogy integration, emphasizes the necessity for educators to incorporate AI into their instructional practices to facilitate and oversee student-centered learning experiences. The fifth competency, AI’s role in enhancing organizational learning, suggests that educators should utilize AI tools effectively to tailor and improve their engagement in professional learning communities.

The third level Create outlines the advanced competencies necessary for educators to effectively design artificial intelligence systems and implement AI innovatively within educational contexts. This includes leadership in developing ethical guidelines, customizing AI systems for specific local needs, and pioneering pedagogical applications of AI that promote social justice, inclusion, and human development. ^59^ The initial competency, social responsibility, emphasizes that educators must engage in the advancement of inclusive AI societies by critically analyzing the impact of AI on social norms and advocating for AI design and implementation that fosters human welfare, inclusion, and social justice. The second competence, co-creating ethical rules, emphasizes that teachers should advocate for AI ethics by facilitating discussions and actions that consider the ethical, social, and environmental implications of AI use, as well as by participating in the development of ethical guidelines for educational AI practices. The third competency, creating with AI, emphasizes that educators must exhibit the capability to adapt AI tools, utilizing both advanced conceptual and practical knowledge to foster inclusive, AI-enhanced learning environments and tackle wider educational issues. The fourth competency, AI-enhanced pedagogical innovation, emphasizes the necessity for educators to critically assess the influence of AI on education. It involves the design of AI-integrated lessons aimed at developing students’ subject-specific, interdisciplinary, critical thinking, and problem-solving abilities, as well as the utilization of data and feedback to further investigate student-centered pedagogical advancements. The fifth competency, AI to support professional development, emphasizes that educators can customize and adapt AI tools to address their professional learning requirements, consistently evaluating and validating AI-supported strategies that improve both their personal growth and that of their professional communities.

According to ^59^ the three progression levels provide a step by step framework for teachers’ profesional growth. The competencies outlined indicate that educators must have both a technical understanding for evaluating AI tools and an ethical disposition, along with reflective capacity, to assess these tools. They should also question the social implications of AI usage, manage associated risks, and safeguard students from algorithmic bias. Teachers, through responsible oversight, ensure that AI-enhanced gamification does not transform into a tool for automated decision-making but instead retains a human-centered pedagogical design that fosters critical thinking, inclusion, and professional development.

## From motivation to transformation: Deep gamification and AI in 21st-Century education

Gamification has demonstrated efficacy in education by enhancing student motivation. ^61^ It is not uniform, necessitating a distinction between two primary approaches: superficial gamification and deep gamification.

Although gamification, conceived as a methodological strategy ^62^ or as the incorporation of game elements into non-game contexts, ^63, 64^ has demonstrated its effectiveness in influencing human behavior, when limited to reward systems, its pedagogical potential is reduced, turning it into a practice centered solely on students’ superficial engagement. Therefore, it is essential that future teachers design gamified proposals that stimulate both intrinsic and extrinsic motivation and receive training that enables them to develop approaches capable of transcending external stimuli.

Based on this conceptual distinction, various studies such as ^35, 65– 68^ have identified key differences between shallow and deep gamification. While the former is limited to external reward mechanisms, such as points or leaderboards, the latter integrates immersive elements and pedagogical decisions aimed at generating meaningful experiences. ^66^ These differences encompass aspects such as pedagogical approach, the type of motivation, the impact on learning, design complexity, and the teacher’s role in the process. According to, ^35^ there are eight fundamental differences between shallow and deep gamification.

Firstly, regarding the teaching and learning process, del Olmo-Muñoz et al. ^69^ indicate that shallow gamification does not lead to substantial changes, whereas deep gamification introduces significant transformations in educational dynamics. Secondly, in terms of implementation difficulty, Mozelius ^66^ points out that shallow gamification is easy to apply, unlike deep gamification, which requires complex planning. Third, concerning pedagogical approaches, Hwang et al. ^68^ explain that shallow gamification is limited to structuring learning, whereas deep gamification directly intervenes in content. Fourth, with respect to the elements used, Mozelius ^66^ highlights that shallow gamification relies mainly on external rewards, in contrast with the meaningful and immersive game mechanics that characterize deep gamification. Fifth, Gurjanow et al. ^65^ underscore that shallow gamification requires technical skills such as programming and graphic design, whereas deep gamification demands expertise in game design with a pedagogical focus. Sixth, regarding motivational impact, the same authors state that shallow gamification produces limited effects, unlike the deep and lasting impact of deep gamification. Seventh, in terms of types of motivation, Mozelius ^66^ differentiates that shallow gamification fosters extrinsic motivation, whereas deep gamification promotes students’ intrinsic motivation.

Ultimately, regarding the duration of involvement, Söbke ^70^ suggests that superficial gamification is typically short-lived, but profound gamification persists over an extended period.

The differences between shallow and deep gamification reveal that gamification can offer different levels of depth, impact, and educational purpose. Accordingly, in order to achieve more elaborate gamified activities with sustainable effects over time, it is fundamental to integrate elements that foster students’ intrinsic motivation. ^71, 72^ In this regard, Turan et al. ^73^ highlight the importance of designing gamified experiences that not only use rewards but also promote meaningful transformations in educational processes.

A crucial distinction to clarify is the difference between deep gamification and AI-driven personalization. While these two concepts are frequently addressed in conjunction, they embody fundamentally distinct constructs. Deep gamification involves the incorporation of significant and engaging game elements, including narrative, missions, collaboration, exploration, and formative feedback, which alter the fundamental framework of a learning activity to enhance intrinsic motivation, autonomy, competence, and relatedness. ^65, 66^ The objective is to alter the manner in which learners create meaning and interact with knowledge.

Conversely, AI-driven personalization refers to a data-driven adaptive mechanism in which artificial intelligence algorithms tailor content, difficulty, sequencing, and feedback to each student’s cognitive profile, behavioral patterns, preferences, and emotional state. ^37, 51, 52^ Whereas deep gamification is grounded in pedagogical intentionality and narrative coherence, AI-driven personalization focuses on optimization and adaptation through continuous analysis of students’ data.

**Figure 2.**
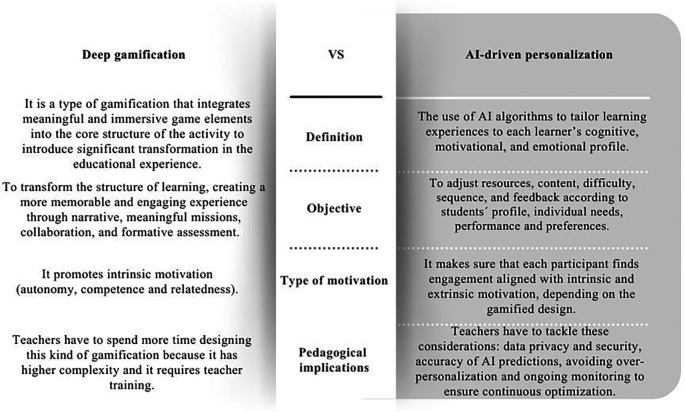
Comparative table on the differences between deep gamification and AI-driven personalization. Note: This figure presents the differences between Deep gamification and AI driven personalization. Own creation based on Refs. 40, 47, 51, 52, 65, 66, 69, 70.

Understanding this distinction is essential to avoid confusing the pedagogical depth of gamification with the technical capacities of AI. As illustrated in Figure 2, which summarizes these conceptual differences and outlines their pedagogical implications, deep gamification transforms learning through meaning, narrative, and intrinsic motivation, whereas AI-driven personalization enhances adaptability and individualized support.

In this context, recent research has explored gamification and identified both advantages and challenges associated with its implementation. For example, Dah et al. ^74^ warn that one of the main challenges of gamification is the prevalence of the “triad of badges, points, and leaderboards,” which fosters only extrinsic motivation and superficial engagement. While shallow gamification can spark interest through playful elements such as points and rewards, it does not significantly transform the learning experience. ^63, 75, 76^ Its effects are often ephemeral ^66, 77^ and tend to diminish in effectiveness when students lose interest in rewards, progressively reducing the effectiveness of these stimuli. ^78– 80^ Given these limitations, it is necessary to rethink gamification in the 21st century by incorporating elements that enhance intrinsic motivation and foster meaningful transformations. ^71– 73^ Some studies emphasize that deep gamification can generate radical changes in teaching–learning processes by integrating game mechanics into the core structure of the activity and creating narrative and immersive experiences that increase intrinsic motivation. ^77^

According to, ^40^ AI can strengthen the design of deep gamification experiences by generating personalized learning, providing immediate feedback, and creating more engaging interactive environments. From this perspective, artificial intelligence becomes a tool that allows teachers to redesign gamified experiences with greater pedagogical depth.

In this context, artificial intelligence constitutes a resource capable of reinforcing deep gamification by helping teachers design gamified environments in a dynamic, adaptive, and student-centered way, offering personalized learning, immediate feedback, and more attractive interactive experiences. ^40^ However, the implementation of this type of gamification faces challenges related to time, pedagogical planning, alignment with learning objectives, and scalability. ^65^

After establishing the relevance of integrating artificial intelligence into gamification designs, explaining how this combination fosters 21st-century skills, and highlighting the need for deep gamification experiences supported by AI, this opinion article argues that the integration of artificial intelligence into gamification processes can strengthen the design of deep gamification experiences by enabling highly personalized learning, adapted to students’ pace, styles, and needs, with immediate feedback and more engaging interactive environments.

The following parts provide the arguments and evidence that are in favor of the assertion that is made in this article.

## AI and deep gamification: A combination for personalized learning

After talking about how important it is to develop deep gamified settings, we need to look more closely at how the combination of deep gamification with artificial intelligence makes individualized experiences that fit each student’s needs, pace, and learning style.

Since educational systems must focus on the learner, personalizing earning is no longer simply a pedagogical goal but an urgent necessity. It is worth noting that, according to, ^51, 81^ artificial intelligence, when integrated into deep gamification designs, opens new possibilities for adequately adapting learning environments to the particularities of each student.

According to studies on deep gamification and the use of AI, this integration allows learning experiences to be personalized by adapting challenges, rewards, and feedback according to student performance, interests, and learning pace, thus fostering both interactive and motivating learning. ^47, 51, 52, 82^

Furthermore, according to, ^36, 83^ the synergy between AI and gamification allows the configuration of personalized educational experiences by analyzing student behavior in real time and dynamically adapting elements of the gamified environment, such as difficulty level, feedback, and rewards.

In contrast to some platforms that merely offer external rewards or standardized mechanics in shallow gamification designs, AI facilitates the creation of truly personalized experiences. Therefore, deep gamification enhanced by AI transcends playful interaction and becomes a powerful methodological strategy for personalizing learning in different educational contexts.

With respect to scaling and challenge adjustment, AI algorithms adjust task difficulty in real time, given that, according to, ^37, 52^ AI not only automates processes but also allows gamified elements (levels, challenges, rewards, and feedback) to be dynamically adapted based on students’ profiles and individual progress. In addition, according to, ^84^ generative artificial intelligence can accurately identify students’ misconceptions and use this information to generate appropriate feedback that addresses their learning needs.

Moreover, Markauskaite et al. ^85^ emphasize that AI becomes a valuable resource for teachers to optimize their time and focus on other important aspects of teaching, such as creating more complex and deeper learning opportunities. However, Giannakos et al. ^21^ stress the importance of teachers critically evaluating AI-powered feedback and complementing it with their own expertise.

This personalization capacity provided by AI aligns with the principles of deep gamification by facilitating immersive, meaningful learning experiences geared toward intrinsic motivation. According to, ^40^ through adaptive narratives and contextualized challenges, authentic and sustained student engagement can be promoted.

An example that illustrates the importance of personalization in the design of meaningful and culturally relevant linguistic experiences is the study by Xia et al. ^86^ In their research, the authors present the Intercultural Language Learning Intelligent System (CILS), which employs artificial intelligence to provide learning experiences tailored both to students’ profiles and to their cultural context. The results also highlight that adjusting teaching and learning strategies according to students’ cultural backgrounds and individual characteristics not only improves the effectiveness of the learning process but also fosters inclusive and meaningful communication in language learning environments.

Another example that demonstrates how the combination of artificial intelligence and gamification can personalize the level of difficulty, offer adaptive feedback, and track student performance in real time is the study by Laverde-Albarracín et al. ^87^ This research implemented a teaching strategy that integrated gamified resources and AI algorithms to generate automatic feedback and adapt challenges according to each student’s performance. AI tools were also used to monitor response time and accuracy in solving exercises, enabling personalized adjustments. Notably, results revealed a 40% increase in the resolution of complex problems in the experimental group compared to the control group.

Similarly, the study by Mohammed and Jesudas ^88^ shows how the integration of AI and gamification transforms language learning. AI offers personalized learning paths, immediate feedback, and content adapted to individual needs, while gamification fosters engagement and motivation through dynamic experiences. This study concludes that the combination of AI and gamification promotes an interactive learning environment that strengthens students’ language skills, sustains motivation and engagement over time, reinforces learning through gamified repetition, generates immersive, contextualized, and culturally relevant experiences, encourages collaboration and competition, and facilitates autonomous learning.

Furthermore, Liu, ^52^ Cabrera Félix and Román Santana ^89^ highlight that incorporating artificial intelligence into gamified environments allows to implement personalized pedagogical approaches tailored to students’ individual needs, while also fostering interaction and generating meaningful educational experiences.

Moreover, Bachiri et al., ^38^ Kassenkhan et al., ^47^ Pardim et al., ^83^ Martínez et al., ^90^ Pelletier et al. ^91^ demonstrate that AI-enhanced gamified designs not only increase students ’participation but also improve feedback and adapt activities based on students’ performance and individual characteristics. These studies reinforce the idea that integrating AI into gamified design is an effective strategy to personalize learning, stimulate sustained progress, and address classroom diversity.

Likewise, Abbes et al. ^51^ argue that incorporating artificial intelligence into gamified environments designed by teachers offers an opportunity to transform how students learn, as it makes it possible to create unique learning paths for each student, responding to their individual needs and stimulating continuous learning progress.

In this context, it is essential to consider students’ diverse learning styles (visual, verbal, active, reflective, and sensory) when designing AI-supported gamified environments. ^92^ Adequate alignment between challenges and learning objectives with students’ interests and styles fosters active participation and stimulates problem-solving skills, allowing each student to progress at their own pace. ^93, 94^ Thus, teachers’ identification of these learning styles enables AI to contribute to the creation of personalized resources and the consolidation of deep gamification experiences. ^95^

In addition, artificial intelligence, by efficiently filtering and organizing large volumes of information, facilitates teachers’ ability to make relevant pedagogical adjustments, adapting both teaching methods and resources to students’ individual characteristics. ^96^ Platforms such as *Classcraft*, *MathCityMap*, and *@MyClassGame* provide concrete examples, as they allow for the implementation of both shallow gamification strategies, based on points, badges, and leaderboards, and deep gamification strategies focused on narratives, meaningful missions, and collaborative dynamics. In this sense, it is up to teachers, based on their formative objectives, to determine which approach is the most relevant. ^57^

Although research evidence supports the potential of AI in gamification designs to improve personalization of learning, it is important to stress that, according to, ^67^ pedagogical creativity and decision-making remain the responsibility of the teacher, since the teacher plays an important role in the personalization process and trains the algorithms to obtain better results.

Finally, it’s important to talk about the problems that come with customisation. The research conducted by Gao et al. ^78^ concluded that the customization algorithms in the AI-enhanced gamified platform ShouTi Fitness require refinement to ensure that recommendations are tailored to students’ proficiency levels, as 10% of participants said that certain suggestions were redundant. Moreover, Hallifax et al. ^58^ caution that modifying game aspects to correspond with students’ psychological profiles presents an ethical quandary: personalized gamification may exploit learners and curtail their agency. In the same way, too much adaptation might make pupils only interact with things they are already comfortable with, which can slow their growth and make it harder for them to deal with new problems.

## AI and deep gamification: An innovative alliance with real impact on learning

As noted in the previous section, the application of AI in education not only personalizes learning within gamified designs but also opens new possibilities for the development of teaching resources, the creation of interactive environments, and the comprehensive transformation of teaching and learning processes. ^51^

In this regard, several studies have shown that the integration of AI improves the efficiency and adaptability of teaching activities, while enabling dynamic, meaningful, and student-centered learning experiences. ^37, 68^ These findings reinforce the idea that when AI is articulated with methodological strategies such as deep gamification, it not only facilitates the personalization of learning but also generates a real and sustainable impact on educational outcomes.

For example, Bennani et al. ^37^ highlight that incorporating gamified challenges and interactive components into immersive educational environments optimizes academic performance, fosters motivation, and stimulates creativity. They also propose integrating artificial intelligence to facilitate students’ access to information, taking into account individual characteristics and profiles.

Similarly, Erbaşı et al. ^81^ argue that the convergence of AI and gamification produces a radical change, as it enables the design of efficient and meaningful learning experiences. Essentially, this union represents an effective set of tools to develop engaging learning systems with better academic results.

Studies on the combination of AI and gamification, such as, ^39, 40^ agree that integrating artificial intelligence into deep gamified environments constitutes an innovative strategy to transform learning. The following section addresses why this innovative alliance should be seriously considered.

Recent empirical evidence expands this perspective. Gao et al. ^78^ note that the synergy between deep gamification and artificial intelligence increases students ‘motivation and engagement by creating interactive environments in which learners are actively involved in their learning process. Gamified designs stimulate exploration, decision-making, and problem-solving in playful contexts, while AI enriches these experiences with personalization, adaptive feedback, and real-time analysis. In this way, meaningful learning and autonomy are strengthened, and academic performance is improved. ^52, 67, 97^

The study by Liu ^52^ provides complementary evidence. With a sample of 486 university students from an English teaching program in China, it compared three AI-powered gamification strategies: adaptive learning paths, conversational agents, and interactive storytelling. The results showed that the combination of gamification and AI increased motivation, personalized the learning experience, and enriched learning, particularly through interactive storytelling that incorporated diverse cultural contexts.

Specifically, students valued the immediate feedback from conversational agents, the cultural richness of interactive storytelling, and the personalization of adaptive learning paths. Scoreboards, unlockable content, and individualized challenges sustained interest and reinforced intrinsic motivation, which translated into significant improvements in both language proficiency and student engagement. In contrast, the control group, which followed a conventional course without AI or gamification, showed no significant progress, confirming that the deep integration of AI and gamification transforms the learning experience by making it more meaningful, motivating, and personalized.

Although the study by Liu ^52^ is not explicitly framed within a critical pedagogy perspective, the inclusion of cultural elements into interactive storytelling can be interpreted as an attempt to link learning to meaningful contexts for students. This aspect aligns, at least partially, with the arguments of Teräs, ^6^ Williamson, ^7^ Facer, ^8^ who emphasize the importance of addressing the realities and needs of local communities.

On the other hand, artificial intelligence facilitates teachers’ work, since in deep gamified environments AI allows real-time analysis of students’ behavior and progress, automatic adaptation of levels and rewards, and identification of learning styles and emotional states. These capabilities, according to, ^96, 98, 99^ enable teachers to focus on the pedagogical dimension, intervene in a timely manner, and address students’ needs in a personalized way.

### AI in action: Crafting a deep gamified experience

**Figure 3.**
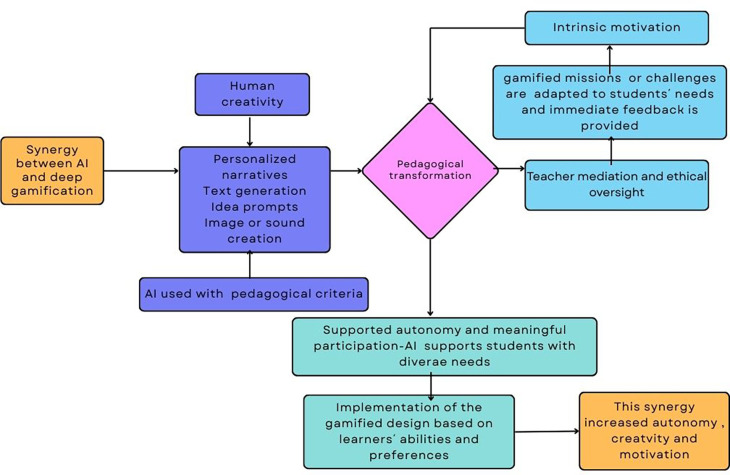
Diagram about how AI supports deep gamification. Own elaboration based on Mèndez Cabrera and Fajkisovà ^100^ study.

This subsection presents an empirical example that demonstrates how artificial intelligence can effectively support deep gamification in authentic educational settings. This example, complemented by Figure 3, shows how AI contributes to narrative enrichment, adaptive challenge design, multimodal resource creation, and inclusive participation, elements that characterize deep gamification and reflect the pedagogical principles discussed in earlier sections.

The study by ^100^ illustrates how AI can meaningfully support deep gamification in real educational contexts. In their study, AI enabled the personalization of narrative elements, contributed to content creation, provided immediate feedback, and scaffolded the writing process. AI tools adapted outputs to each student’s linguistic level, creativity, and personal preferences, making the experience both inclusive and authentic. Within a shared science-fiction narrative, students collaboratively developed characters, plots, and multimodal artefacts such as texts, images, and sounds, using generative AI tools including ChatGPT, Bing Image Creator, Leonardo, and Donna. The gamified experience integrated narrative missions, teamwork, and formative feedback, allowing each student to co-create within a common story world. It’s worthy to highlight that the design fostered inclusion by enabling students with diverse abilities and learning needs to participate actively and express themselves through modalities aligned with their skills and interests. Throughout the process, teachers guided the process, ensuring ethical AI use, and maintaining a clear focus on pedagogical goals rather than technological novelty.

This project exemplifies a deep gamification design because it incorporates game elements such as narrative immersion, adaptive missions, and collaboration within a meaningful, student-centered pedagogical framework. Instead of relying on external rewards, the gamified system fostered intrinsic motivation, autonomy, and reflection by transforming learning into a creative quest. Artificial intelligence was integrated into this design and it allowed to make the experience both inclusive and authentic. The synergy between AI and deep gamification therefore supported autonomous, meaningful, and multimodal learning, illustrating how technology can enrich education when guided by sound pedagogical principles (see Figure 3).

In summary, the combination of deep gamification and artificial intelligence entails significant implications for teaching practice and the optimization of teaching and learning processes at all educational levels. In this sense, teachers should rely on AI to design gamified environments that respond to students’ interests and needs. ^53^ This requires moving toward deeper pedagogical proposals that integrate meaningful narratives, adaptive feedback, and contextualized challenges, overcoming mechanical models focused exclusively on extrinsic rewards. ^100, 102^

Similarly, it is imperative that students engage actively in their educational journey, fostering the growth of autonomy and decision-making abilities. Strategies like creating personalized avatars or customizing aspects of the gamified environment can enhance identification, boost engagement, and reinforce commitment to learning, so promoting intrinsic motivation.

The utilization of gamification analytics technologies, such as GamAnalytics, in AI-enhanced gamified environments enables educators to more precisely track student interactions with gamified systems. By visualizing and analyzing learning data produced by AI, such as speech, gestures, and student action logs, educators receive feedback to modify game dynamics and enhance gamification designs, resulting in increased engagement, motivation, and learning outcomes. ^21, 103^ This establishes a co-creation process between educators and artificial intelligence, designed to enhance the educational experience and revolutionize teaching and learning methodologies.

## Between potential and the gap: Ethical and technological challenges of AI-optimized Gamification

Despite the immense pedagogical potential of the combination of artificial intelligence and profound gamification, its implementation presents substantial challenges that must not be disregarded. The following are particularly noteworthy among these:

The first challenge concerns data protection, ethics, and privacy. Gamification mediated by artificial intelligence collects large volumes of information about students, including their academic progress, in-game behavior, preferences, and even demographic data, which poses risks if the security and confidentiality of such records are not guaranteed. ^104, 105^ To address this issue, it is essential to establish strong security measures such as data protection protocols and audits of AI models to detect bias and prevent misuse. ^38, 40^

The second challenge has to do with avoiding excessive dependence on technology. AI should be understood as a means and not an end in educational processes. ^96^ Therefore, it is not advisable to fully delegate the design of gamified experiences to artificial intelligence, especially regarding content personalization, the difficulty of challenges and narratives, since game mechanics must always respond to pedagogical objectives. ^40, 106^ In this sense, AI should play a complementary role, contributing to analysis, monitoring, and optimization, while pedagogical design remains the responsibility of the teacher. ^107^

The integration of AI into deep gamification requires a pedagogically grounded approach that balances innovation with caution, ensuring that adaptive and generative technologies enrich the learning experience. Based on the ^59^ AI Competency Framework for Teachers, the fourth competence called *AI pedagogy integration* of the Deepen level provides a balanced model for reconciling AI’s transformative potential with the risks of over-dependence because according to this competency teachers should skillfully integrate AI into designing and guiding student-centered learning to foster engagement, support individualized instruction, and improve teacher-student interactions with the purpose of promoting critical thinking, problem-solving skills, and empathy among students. This competency involves evaluating AI tools pedagogical affordance for student-centric pedagogical activities, in this case for gamified designs, cooperating with peers or experts to assess whether the design of generative AI systems takes into account pedagogical implications, designing student-centric teaching and learning activities based on validated educational AI tools and avoiding the use of AI automate the design, administration and grading of assessments by analyzing the risks of AI in usurping human responsibility when providing feedback and making decisions on students’ learning outcomes.

The third challenge relates to the technological and infrastructural gap. Significant inequalities persist in access to, use of, and proficiency with information, communication, and artificial intelligence technologies. ^68^ UNESCO, ^44^ in its 2023 agenda, stresses that young people must be guaranteed access to both formal and informal educational experiences that broadly integrate technology to ensure greater equity in opportunities.

This gap affects both teachers and students who lack technological resources in their educational institutions. ^88, 90^ Neubaum et al. ^108^ showed that during the pandemic, advances in digital skills mainly benefited young people with greater resources, thus deepening inequalities rather than reducing them. According to, ^96^ this exclusion stems from social, demographic, and educational factors that limit equitable access to the benefits of technological advances.

With respect to infrastructure, the need to provide educational institutions with both physical and digital resources has become evident: devices, connectivity, educational software, digital platforms, and tools for developing AI-supported gamified experiences. ^90^ The lack of such resources restricts the effective implementation of new proposals, especially in developing countries where investment in technological equipment remains insufficient. ^96^

The fourth challenge involves the cultural and pedagogical appropriation of technology. As Feenberg ^109^ argues, technologies are not neutral, but neither are they closed: they can be adapted and reconfigured according to context. However, as Watters ^110^ warns, the problem does not lie in the rigidity of technology but in the uncritical adoption of external models, particularly from the Global North, without adapting them to local contexts, languages, and teaching practices. De Sousa Santos and Meneses ^111^ reinforce this idea, by pointing out that it is not about incorporating tools without reflection but about questioning their meaning within situated educational processes. Instead of replicating foreign uses, it is more relevant to start from our own educational and cultural needs. Innovation, in this sense, should be conceived as situated creation rather than mere imitation.

The fifth challenge relates to teacher training, a fundamental aspect for harnessing the potential of AI-mediated deep gamification. According to ^51^ the lack of specialized preparation significantly limits its impact in education. Dah et al. ^74^ agree that the effectiveness of gamification depends on multiple factors: design quality, ^112^ theoretical grounding, ^113, 114^ standardization of elements, ^113^ individual differences, ^115^ and contextual particularities. ^116^ These conditions underscore the need for teachers trained in gamification software, ^117^ capable of adapting resources to their educational contexts and to the characteristics of their students. ^118^

In order to progress toward meaningful implementation, it is imperative to provide training to both in-service and preservice teachers on the development of gamified experiences that incorporate AI as a support tool. This training should encompass the acquisition of critical digital literacy skills, the comprehension of adaptive systems, and the mastery of resources such as immersive narratives, dynamic feedback, and contextualized challenges.

Finally, despite the challenges, the future of AI-supported gamification is promising. This technological convergence can transform learning into a more dynamic and motivating experience by encouraging student participation and allowing the continuous adaptation of gamification designs throughout the learning process. ^103^ In this context, addressing current challenges from a critical and proactive perspective will make it possible to fully leverage the transformative potential of AI in deep gamification environments. Ultimately, true innovation, as Örpek et al. ^36^ argue, will depend on whether educational institutions and educators manage to balance the intelligent use of technology with situated pedagogical objectives, ensuring that educational practices respond to the needs of local communities.

## Conclusions

This opinion piece critically examines how the use of artificial intelligence might improve deep gamification ideas. This synergy goes beyond approaches based on external rewards and opens the door to a pedagogical transformation centered on the creation of authentic, adaptive, and motivating experiences that foster meaningful learning, stimulate intrinsic motivation, and strengthen students’ commitment to their own learning process.

The analysis underscores the significance of acknowledging that artificial intelligence does not supplant the teacher’s function but rather augments it by providing resources for personalization, real-time feedback, and automated material development. The gamification of educational experiences relies on the educator, who, from a critical standpoint, crafts narratives and challenges while considering the reality, interests, needs, and learning styles of their pupils.

The reflection also makes it clear that combining AI and deep gamification is not only a technological upgrade; it is a change in the way we talk about human and artificial intelligence. This change means that teachers need to create meaningful learning experiences instead of just giving students fun activities to do to keep them interested in class. ^119^ They must also revise their pedagogical approaches to facilitate the acquisition of 21st-century competencies, while addressing ethical concerns such as privacy, technological equity, and cultural relevance. The effective incorporation of AI to improve deep gamification relies on educators acquiring specific skills that allow them to critically analyze algorithmic results, navigate ethical dilemmas, and make educated teaching choices. In this regard, AI-enhanced deep gamification may realize its revolutionary potential solely when anchored in a human-centered, pedagogically deliberate, and ethically accountable framework.

Ultimately, it is crucial to perceive artificial intelligence as a pivotal instrument for converting static gamification into dynamic, adaptable, and learner-centered educational experiences. ^37^ The revolutionary potential will be actualized alone via deliberate planning, robust teacher training, and the ethical and contextual application of technology. Thus, profound gamification enhanced by artificial intelligence would facilitate authentic educational transformations in the contemporary period.

## F1000 AI policy

We have read and agree to comply with the F1000Research AI Policy. We confirm that, in accordance with this policy, ChatGPT-5 was used for style correction review. In addition, generative AI tools were employed in the initial design of the figures included in this article, which were subsequently refined, edited, and validated by the authors to ensure accuracy and alignment with the manuscript’s objectives. All uses of generative AI were conducted under the supervision of the authors, with full transparency and rigorous review.

## Data Availability

No data are associated with this article.
