# Deep Gamification and Artificial Intelligence as Catalysts of Educational Transformation

**DOI:** 10.12688/f1000research.171453.3

**Published:** 2026-04-24

**Authors:** Diana Milena Patiño Barriga, Ana Dolores Vargas Sánchez, Paloma Valdivia Vizarreta

**Affiliations:** 1 Universidad de La Sabana, Chia, Cundinamarca, Colombia; 2 Universitat Autonoma de Barcelona, Barcelona, Cataluña, Spain

**Keywords:** Gamification, artificial intelligence, learning, innovation, social transformation

## Abstract

This opinion article examines the convergence between artificial intelligence (AI) and gamification in learning environments, with an emphasis on deep gamification designs aimed at creating interactive and meaningful experiences that transcend extrinsic motivation. The introduction sets the context by presenting AI as a tool that must be critically analyzed within the framework of critical pedagogy, which underscores the importance of adopting technology reflectively, placing the student at the center, and responding to the real needs of the community. The body of the article develops this claim: the integration of artificial intelligence into deep gamification designs can contribute to genuine educational transformation, provided that such integration is guided by critical pedagogical principles and maintains a balance between the potential of technology and the formative power of teaching practices. Arguments related to this claim are provided, including the use of AI in gamified designs to create personalized learning experiences based on students’ needs, rhythms, and learning styles; to transform the way students learn; to offer new educational resources; to adapt elements such as difficulty levels, challenges, and feedback in real time; and to develop engaging learning systems that lead to better academic outcomes. The conclusion emphasizes the significance of instructors utilizing critical pedagogy to direct AI-optimized gamification designs by incorporating culture, creativity, and critical awareness as pillars of educational transformation. This approach enables the surmounting of the obstacles presented by this synergy.

## Introduction

In the 21st century, the integration of digital technologies into modern education has been characterized by narratives advanced by political and corporate entities, as well as certain multilateral organizations, advocating for education to be congruent with the requirements of a globalized and digitized marketplace. ^1^ ^,^ ^2^

From a critical and contextual pedagogical viewpoint, education constitutes a multifaceted social practice imbued with historical, ethical, and political significance, aimed at cultivating critical individuals and reinforcing the social fabric. ^3^ ^,^ ^4^ Therefore, it is important to note that the educational function should not be reduced to a merely adaptive response to technological change. ^5^

The subordination of pedagogical objectives to technological means is one of the most persistent tensions in contemporary educational discourse. A narrative has emerged that prioritizes technology as the primary driver of educational transformation, while relegating pedagogical reflection to a secondary position, all in the name of efficiency, innovation, and competitiveness. ^6^ ^,^ ^7^ In this context, teacher training is depicted as a continuous and expedited updating process, necessitated by technological urgency, rather than as a critical exercise associated with the essence of teaching. ^8^

Pedagogy should take a critical view of technology, according to ^4^ ^,^ ^9^ instead of using technologies made in other countries, often by companies with goals other than education, the educational system should create technologies based on local knowledge and community needs. This point of view shows that the value of pedagogy is not in how well it works technically, but in building meaningful relationships with knowledge, encouraging critical thought, and taking care of the connections between people and communities. ^3^

The COVID-19 pandemic has illustrated that it is not the technological tools themselves that support educational processes; rather, it is the pedagogical knowledge, the contextualized use, and the ability of teachers to reorganize practices and adapt available tools in a creative, appropriate, and socially meaningful manner, based on the needs of their students. ^10^ Therefore, this demonstrates that it is not technology itself that transforms education, but rather its critical, contextual, and relational appropriation, ^11^ ^–^ ^13^ which is accomplished in the classroom through the competencies that teachers develop. In this context, facing the widespread demotivation of 21st-century students, who no longer learn or engage in the same way as previous generations, ^14^ ^–^ ^16^ teachers have resorted to various methodological strategies, among them gamification, to increase student motivation and engagement. ^17^

At the same time, education is being influenced by the integration of new technologies such as artificial intelligence (AI), which have the potential to support more interactive learning environments. ^18^ Nonetheless, the incorporation of these emerging technologies requires situated, conscious pedagogical decisions, constructed in dialogue with the real needs of each educational community. ^10^ ^,^ ^19^ ^,^ ^20^

Moreover, this potential must be critically examined, since educational innovation not only involves incorporating technologies such as generative artificial intelligence to create interactive environments like gamified platforms, but also articulating these tools with pedagogical approaches capable of responding to the challenges of inclusive, relevant, and ethical education. ^21^

The opinion article aims to analyze how the integration of artificial intelligence into deep gamification designs may contribute to authentic learning experiences, provided that such integration is guided by critical pedagogical principles and maintains a balance between the potential of technology and the formative power of teaching practices. In this sense the study adopts a thoretical and integrative perspective, aiming to contribute to ongoing scholarly discussion rather than to provide empirical evidence.

## Method

This opinion article is grounded in a thematic conceptual synthesis supported by a conceptual review and a critical synthesis of the literature on artificial intelligence, gamification and critical pedagogy. The purpose of the analysis was to examine and compare key scholarly contributions, and to identify conceptual patterns, convergences, and tensions across recent and relevant sources drawn from studies addressing the integration of deep gamification and AI in recognized databases such as Scopus and Web of Science.

The analytical process followed an iterative and interpretive procedure. First, relevant publications were selected based on their conceptual contribution to understanding deep and shallow gamification, AI-supported learning environments and the development of 21st-century skills. Second, these texts were read closely to extract significant ideas, pedagogical arguments, and recurring constructs. Our review reported that, despite the growing importance of artificial intelligence and gamification in education, few studies explore their intersection, particularly within the context of deep gamification. Third, units of meaning were coded and clustered, allowing higher-order conceptual themes to emerge. This process resulted in six major categories that structure the argument developed in this article: deep and shallow gamification as a framework to understand AI integration, synergies between gamification and pedagogy, benefits of integrating AI into deep gamified designs, risks of incorporating AI into gamified designs, the role of teachers within AI-optimized gamification, and ethical and technological challenges (see appendix).

Throughout the article, we include examples from current research to illustrate how AI supports deep gamification through personalization, adaptative challenges, real-time feedback, and immersive narratives. This method privileges conceptual clarity and integrative synthesis over exhaustive coverage, aligning with the reflective purpose of this opinion article.

## Three emerging synergies between AI, gamification, and pedagogy for the development of 21st-century skills

**Figure 1.**
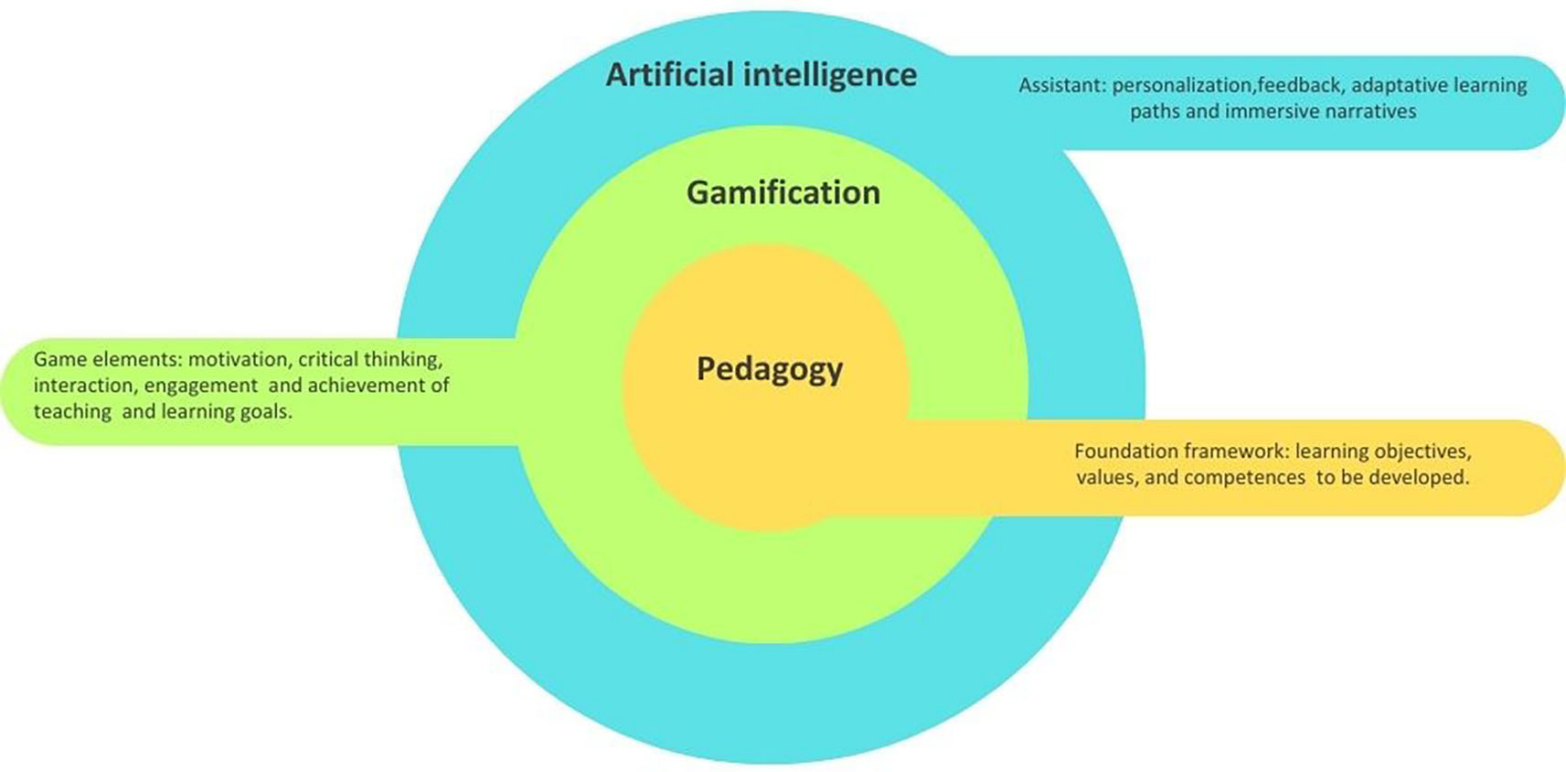
Relationship between pedagogy, gamification and artificial intelligence. Note: The figure illustrates a three-layer model in which pedagogy forms the core foundation of teaching and learning; gamification emerges as an intermediate layer integrating game elements to transform traditional learning environments and artificial intelligence functions as an outer assistant layer enabling personalization, adaptative pathways, and immersive narrative design. Own source elaborated, adapting the multi directional model of pedagogical reasoning for gamification ^23^ and the AI oriented pedagogical model. ^22^

The relationship between pedagogy, gamification and artificial intelligence is fundamentally interdependent. Rather than functioning as separate components, they interact to shape how students engage, participate, and learn in contemporary learning environments (see Figure 1). In the three-layer model, pedagogy occupies the central position because it provides the foundational core that defines learning objectives, values, and competencies. It involves the decisions teachers make about how students learn most effectively within specific contexts and emerges from the relational interactions between teachers, students and the learning objects. ^22^

Gamification is positioned in the intermediate layer because it depends on pedagogical reasoning for its meaningful implementation. When grounded in sound pedagogical design, gamification can transform traditional learning environments by fostering motivation, facilitating critical thinking and interaction, engaging students, and supporting the achievement of specific teaching and learning goals. ^23^

Finally, artificial intelligence functions as an external, supportive layer that enhances learning experiences through personalization, immediate feedback, adaptive learning paths, and immersive narratives. Instead of replacing teachers, AI collaborates with them in the co-construction of learning pathways, shifting from a hierarchical, top-down approach to a more dynamic and relational pedagogy. This creates a more collaborative relationship in which educators, students, and AI technologies participate, reestructuring the educational experience. ^22^

Altogether, the three-layer model highlights that AI and gamification only become pedagogically meaningful when guided by intentional, coherent teaching practices. This conceptual foundation enables a deeper understanding of how they interact in real learning contexts. Taking this into account, this section outlines three key synergies through which AI-enhanced gamification can support meaningful educational practices.

From a conceptual perspective, the following synergies are derived from the critical interpretation of recent literatura about arifiial intellugence, gamification and pedagogy.

Synergy 1: Articulating pedagogical knowledge and pedagogical potential

The first synergy suggests that pedagogical knowledge and technological potential must be articulated: AI and gamification provide resources, but it is the teacher who ensures that they make sense in specific educational contexts. Training requires an ongoing dialogue between pedagogy and technology, where artificial intelligence and gamification are integrated from a critical approach that promotes meaningful, active, and contextualized learning. ^24^ From this perspective, educational innovation cannot be limited to the mere incorporation of devices or platforms but must be grounded in coherent pedagogical proposals that give purpose to their implementation. ^25^

To foster meaningful pedagogical innovation, it is essential that artificial intelligence and gamification can be integrated into robust pedagogical frameworks, in which teachers assume a central role as designers and leaders of change-oriented processes. ^26^

From their professional autonomy, teachers promote educational innovation. ^27^ ^–^ ^29^ Their task of validating, contextualizing, and enriching AI-generated content reaffirms their role as facilitators and guides in teaching and learning processes, while integrating the emotional component that technology lacks. ^27^ ^,^ ^30^ ^,^ ^31^

This autonomy should be understood not only as the ability to make important decisions based on professional expertise, but also as the capacity to adapt AI-generated content and supervise the behavior of algorithmic systems and other emerging technologies in education. It involves critically reviewing algorithmic outputs, identifying potential biases, and ensuring that personalization and adaptive feedback remain aligned with students’ needs and cultural contexts. Moreover, it is essential to recognize teachers’ agency in decision-making regarding AI integration, involving them in the design and implementation of institutional and policy frameworks and enabling them to adapt emerging technologies in active and creative ways. ^32^

Thus, the teacher’s role is crucial in designing and developing immersive and interactive experiences that are meaningful and foster sustained engagement among students. ^33^ This responsibility involves contextualizing the incorporation of technology, selecting and re-signifying resources according to students’ needs, pace, and realities, ensuring that innovations do not become ends in themselves but rather means to promote inclusive, critical, and culturally relevant learning. ^34^ ^–^ ^36^

It is important to emphasize that this convergence requires teachers to critically analyze the scope and risks of AI implementation. Based on their knowledge and professional judgment, educators are the ones who lead pedagogical transformation, making decisions about the design, implementation, and continuous improvement of gamified environments.

Synergy 2: AI and gamification as enablers of 21st century skills

The second synergy establishes that when gamification and artificial intelligence are combined, may promote dynamic and motivating learning environments are generated, which may cultivate the development of 21st-century skills. Also, the design of teaching environments can be enhanced integrating AI and gamification, thereby potentially fostering intrinsic motivation, increasing participation, improving academic performance, and promoting the inclusion of students with different learning styles. ^37^ The articulation between the adaptive power of AI and the motivational nature of gamification suggest that they open up new possibilities for. ^35^ ^,^ ^38^

According to, ^39^ this approach may contribute to the development of 21st-century skills such as problem solving, information management, technological appropriation and innovation, ^40^ as well as creativity, communication, collaboration, and critical thinking, recognized by international organizations such as ISTE, ^41^ UNICEF ^42^ OECD, ^43^ and UNESCO. ^44^

Likewise, by integrating playful elements into educational resources, gamification energizes teaching and learning through group activities and case resolution, which foster the 21st century skills mentioned above, ^45^ while also promoting digital learning and technological fluency in interactive, challenging, and contextualized scenarios. ^46^ In this regard, AI-powered gamified educational platforms can design adaptive and personalized experiences, ideal for developing critical thinking through problem-based learning and playful activities adjusted to students’ performance. ^47^

Synergy 3 From skill acquisition to social transformation through critical pedagogy

The third synergy proposes moving beyond the development of skills for individual success to offer students opportunities to transform reality from the perspective of critical pedagogy. In this sense, education should prepare students to become critical thinkers capable of adapting to new knowledge, solving problems, and actively participating in society. ^48^ This vision aligns with critical pedagogy, which emphasizes the importance of students interpreting their contexts, making informed decisions, and generating creative solutions to the challenges they face. Following the approach proposed by Freire 4, this implies fostering tstudents’ capacity to analyze, evaluate, and apply knowledge in conscious, critical, and constructive ways. ^49^

According to, ^50^ only a conscious appropriation of emerging technologies within educational practices allows us to transcend technical novelty and adopt a perspective inspired by Freire’s pedagogy. ^4^

This synergy highlights that the integration of gamification and AI must be grounded in a critical pedagogical stance that empowers students to question, participate, and co-construct knowledge. In a deeply gamified environment enriched with AI, gamified experiences inspired by popular narratives such as *Game of Thrones* can be transformed into powerful opportunities for critical inquiry.

## AI in educational gamification: Between potential implications and emerging risks

After acknowledging the relevance of the three synergies presented above, particularly that of gamification and artificial intelligence as a combination capable of boosting 21st-century skills, it is necessary to analyze in greater detail both the possibilities AI offers to enhance gamification, the risks associated with its implementation and the competences teachers need to develop to face algorithmic bias and ethical oversight.

The integration of artificial intelligence into gamified designs emerges as a strategic reinforcement. Asnoted by Abbes et al., ^51^ its incorporation into education opens new possibilities for the development of innovative teaching resources, may contribute to improving virtual instruction, and provides personalized and adaptive learning experiences that respond to each student´s needs and pace. From this perspective, and in complement to artificial intelligence, gamification plays a key role in the educational process due to its ability to increase motivation, strengthen commitment, and foster students’ autonomy. ^52^ ^–^ ^56^

Moreover, it aids in reinforcing learning by providing dynamic environments that facilitate both repetitive practice and the contextual application of knowledge. As emphasized by García-Martínez et al., ^57^ although gamification itself has numerous advantages in education, its efficacy may be markedly enhanced when combined with the adaptive and personalized features afforded by artificial intelligence.

Nevertheless, it is important to stress that the incorporation of AI into gamified environments also entails risks. Hallifax et al. ^58^ point out that over-reliance on these systems could reduce teachers ‘influence and weaken personal interactions, which are essential for learning.’ Likewise, Liu ^52^ warns that an overload of feedback, interfaces, or complex tasks could overwhelm students, negatively affecting their motivation. Similarly, Hallifax et al. ^58^ emphasize that the implementation of adaptive gamification systems requires advanced infrastructure, accurate models of students’ behavior, and interdisciplinary collaboration, conditions that are not always easy to meet.

The incorporation of artificial intelligence into gamified educational settings necessitates a focus on educator autonomy and ethical supervision, especially as adaptive systems progressively influence classroom dynamics. According to ^59^ AI Competency Framework for Teachers, AI distinguishes itself from earlier iterations of educational technologies by its ability to replicate aspects of human judgment, prediction, and decision-making. This distinctive attribute poses potential risks to human agency, particularly when educators develop excessive reliance on automated recommendations or adaptive learning routes. For AI-enhanced gamification to maintain a pedagogically sound foundation, educators must therefore exercise ongoing interpretive authority, making critical decisions regarding when, how, and to what extent AI-generated feedback or adaptive challenges are consistent with learning objectives and students’ requirements.

A fundamental aspect of instructor autonomy within AI-enhanced environments is the ability to mitigate algorithmic bias. There are various forms of bias in education, such as selection bias, which arises when data do not accurately represent the entire student population; confirmation bias, which involves the tendency to seek information that reinforces existing beliefs; stereotypical bias, which encompasses assumptions based on gender stereotypes; and cultural bias, which manifests through language or examples that are irrelevant to specific cultural contexts. Concerning algorithmic biases, UNESCO ^59^ highlights that exclusion and discrimination may be inherently embedded within AI models and datasets, leading to automated decisions that perpetuate inequalities across gender, language, and socioeconomic status.

Within gamified designs, where performance data influence difficulty levels, reward systems, classifications, and feedback mechanisms, educators must rigorously oversee AI outputs to detect instances of inequitable treatment. This entails evaluating the pedagogical legitimacy of adaptive suggestions, situating automated recommendations within students’ sociocultural contexts, and intervening when gamified feedback inadvertently disadvantages specific groups of students. In this context, educators ensure that AI-enhanced gamification may promote inclusion, diversity, and equitable engagement. ^44^

In relation to ethical oversight, teachers have to rely on robust conceptual frameworks that enable them to make informed, critical decisions about the validity, fairness, and contextual relevance of algorithmic outputs. ^32^ Through this reflective stance, they act as ethical mediators who detect and mitigate algorithmic bias, ensuring that AI-optimized gamification experiences align with pedagogical goals and culturally relevant learning narratives, thus preventing automated systems from losing sight of the educational purpose.

Teachers mediate algorithmic bias by critically evaluating algorithmic outputs. For instance, they apply algorithmic awareness, as a key component of digital literacy, to analyze AI feedback; they ensure equity in learning opportunities by avoiding exclusive reliance on AI-driven ability grouping and by providing support for students who face difficulties; they monitor learning processes in gamified experiences by checking dashboards to identify potential biases in real-time interactions; and they intervene directly in the classroom by deciding whether to keep or modify AI recommendations and by adjusting these suggestions. Moreover, as suggested by, ^60^ it is important to require bias audits and to create communities of practice that allow teachers to share effective strategies for addressing this challenge.

To effectively fulfill the role of ethical mediator and make informed, critical decisions, teachers need to develop specific competencies. According to, ^59^ fifteen competencies required for effective ethical oversight are organized into five dimensions: a human-centered mindset, ethics of AI, AI foundations and applications, AI pedagogy, and AI for professional learning, which are articulated within a three-level progression model. It is important to note that these five competency areas are integrated across every progression level.

The first progression level, *Acquire*, outlines the essential AI literacy that all teachers must develop in order to evaluate, select, and use AI tools appropriately in their teaching practice. This foundational stage includes understanding how AI models are trained, recognizing potential benefits and risks, and identifying ethically relevant issues such as human rights, data privacy, and human- centered design. According to the UNESCO *AI Competency Framework for Teachers*, ^59^ this level comprises five core competencies. The first, human agency, emphasizes that teachers must understand that AI systems are human-created and human-guided, and that the decisions embedded in their design can significantly influence learners’ autonomy and rights. The second, ethical principles, requires teachers to grasp the basic ethical issues surrounding AI and the principles that underpin responsible human–AI interactions. The third, basic AI techniques and applications, involves acquiring foundational conceptual knowledge—what AI is, how models are trained, and how to determine whether particular AI tools are pedagogically appropriate and properly validated. The fourth competency, AI-assisted teaching, expects teachers to identify the pedagogical benefits of AI tools for enhancing lesson planning, instruction, and assessment, while actively mitigating associated risks. Finally, enabling lifelong professional learning highlights the importance of teachers using AI tools to support their own professional growth, reflective practice, and ongoing adaptation to evolving educational demands. ^59^

The initial progression level, Acquire, delineates the fundamental AI literacy that all educators are required to cultivate to effectively assess, choose, and utilize AI tools within their instructional practices. This level encompasses a comprehension of the training processes of AI models, an assessment of their potential benefits and associated risks, and the identification of ethically significant issues such as human rights, data privacy, and human-centered design. According to the UNESCO AI Competency Framework for Teachers, ^59^ this level encompasses five fundamental competencies. The initial principle, human agency, underscores the importance for educators to recognize that AI systems are developed and directed by humans, and that the decisions incorporated into their design can substantially impact students’ autonomy and rights. The second, ethical principles, mandates that educators understand the fundamental ethical considerations related to AI and the principles that underpin responsible interactions between humans and AI. The third, fundamental category of AI techniques and applications, encompasses the acquisition of essential conceptual knowledge, including a comprehension of what artificial intelligence entails, the processes involved in training models, and the criteria for assessing the pedagogical suitability and validation of specific AI tools. The fourth competency, AI-assisted teaching, requires educators to recognize the pedagogical advantages of AI tools in augmenting lesson planning, instruction, and assessment, while proactively addressing and mitigating related risks. The fifth, which facilitates continuous professional development, emphasizes the significance of educators employing AI tools to enhance their ongoing professional growth and reflective practices. ^59^

The second level, Deepen, outlines the intermediate competencies necessary for teachers to develop meaningful pedagogical strategies that ethically and purposefully integrate AI. Teachers at this level must exhibit human accountability in the integration of AI into their lessons, critically evaluate AI tools for ethical implications, ensure safe and responsible usage, promote equity, inclusion, and diversity, comply with legal and institutional frameworks, and apply ethical principles in the identification, selection, evaluation, and implementation of AI tools to improve their teaching practices. ^59^ This level includes five competencies. The first aspect, human accountability, underscores the necessity for teachers to exhibit a comprehensive understanding of human responsibility regarding the appropriate use and implementation of AI. The second principle, safe and responsible use, emphasizes the necessity for educators to internalize ethical guidelines for the responsible and safe application of AI. This includes respecting data privacy and intellectual property rights, which should be applied when evaluating, utilizing, and creating content with AI tools. The third competency, application skills, emphasizes that educators must proficiently utilize AI tools sanctioned for educational use, while systematically integrating ethical principles into their practice. The fourth aspect, AI–pedagogy integration, emphasizes the necessity for educators to incorporate AI into their instructional practices to facilitate and oversee student-centered learning experiences. The fifth competency, AI’s role in enhancing organizational learning, suggests that educators should utilize AI tools effectively to tailor and improve their engagement in professional learning communities.

The third level Create outlines the advanced competencies necessary for educators to effectively design artificial intelligence systems and implement AI innovatively within educational contexts. This includes leadership in developing ethical guidelines, customizing AI systems for specific local needs, and pioneering pedagogical applications of AI that promote social justice, inclusion, and human development. ^59^ The initial competency, social responsibility, emphasizes that educators must engage in the advancement of inclusive AI societies by critically analyzing the impact of AI on social norms and advocating for AI design and implementation that fosters human welfare, inclusion, and social justice. The second competence, co-creating ethical rules, emphasizes that teachers should advocate for AI ethics by facilitating discussions and actions that consider the ethical, social, and environmental implications of AI use, as well as by participating in the development of ethical guidelines for educational AI practices. The third competency, creating with AI, emphasizes that educators must exhibit the capability to adapt AI tools, utilizing both advanced conceptual and practical knowledge to foster inclusive, AI-enhanced learning environments and tackle wider educational issues. The fourth competency, AI-enhanced pedagogical innovation, emphasizes the necessity for educators to critically assess the influence of AI on education. It involves the design of AI-integrated lessons aimed at developing students’ subject-specific, interdisciplinary, critical thinking, and problem-solving abilities, as well as the utilization of data and feedback to further investigate student-centered pedagogical advancements. The fifth competency, AI to support professional development, emphasizes that educators can customize and adapt AI tools to address their professional learning requirements, consistently evaluating and validating AI-supported strategies that improve both their personal growth and that of their professional communities.

According to ^59^ the three progression levels provide a step-by-step framework for teachers’ professional growth. The competencies outlined indicate that educators must have both a technical understanding for evaluating AI tools and an ethical disposition, along with reflective capacity, to assess these tools. They should also question the social implications of AI usage, manage associated risks, and safeguard students from algorithmic bias. Teachers, through responsible oversight, ensure that AI-enhanced gamification does not transform into a tool for automated decision-making but instead retains a human-centered pedagogical design that fosters critical thinking, inclusion, and professional development.

## From motivation to shifting paradigms: Deep gamification and AI in 21st-Century education

Gamification has demonstrated efficacy in education by enhancing student motivation. ^61^ It is not uniform, necessitating a distinction between two primary approaches: Shallowgamification and deep gamification.

Although gamification, conceived as a methodological strategy ^62^ or as the incorporation of game elements into non-game contexts, ^63^ ^,^ ^64^ has demonstrated its effectiveness in influencing human behavior, when limited to reward systems, its pedagogical potential is reduced, turning it into a practice centered solely on students’ superficial engagement. Therefore, it is essential that future teachers design gamified proposals that stimulate both intrinsic and extrinsic motivation and receive training that enables them to develop approaches capable of transcending external stimuli.

Based on this conceptual distinction, various studies such as ^35^ ^,^ ^65^ ^–^ ^68^ have identified key differences between shallow and deep gamification. While the former is limited to external reward mechanisms, such as points or leaderboards, the latter integrates immersive elements and pedagogical decisions aimed at generating meaningful experiences. ^66^ These differences encompass aspects such as pedagogical approach, the type of motivation, the impact on learning, design complexity, and the teacher’s role in the process. According to, ^35^ there are eight fundamental differences between shallow and deep gamification.

Firstly, regarding the teaching and learning process, del Olmo-Muñoz et al. ^69^ indicate that shallow gamification does not lead to substantial changes, whereas deep gamification can introduce meaningful changes in educational dynamics. Secondly, in terms of implementation difficulty, Mozelius ^66^ points out that shallow gamification is easy to apply, unlike deep gamification, which requires complex planning. Third, concerning pedagogical approaches, Hwang et al. ^68^ explain that shallow gamification is limited to structuring learning, whereas deep gamification directly intervenes in content. Fourth, with respect to the elements used, Mozelius ^66^ highlights that shallow gamification relies mainly on external rewards, in contrast with the meaningful and immersive game mechanics that characterize deep gamification. Fifth, Gurjanow et al. ^65^ underscore that shallow gamification requires technical skills such as programming and graphic design, whereas deep gamification demands expertise in game design with a pedagogical focus. Sixth, regarding motivational impact, the same authors state that shallow gamification produces limited effects, unlike the potentially more sustained of deep gamification. Seventh, in terms of types of motivation, Mozelius ^66^ differentiates that shallow gamification fosters extrinsic motivation, whereas deep gamification promotes students’ intrinsic motivation.

Ultimately, regarding the duration of involvement, Söbke ^70^ suggests that shallow gamification is typically short-lived, but deep gamification persists over an extended period.

The differences between shallow and deep gamification reveal that gamification can offer different levels of depth, impact, and educational purpose. Accordingly, in order to achieve more elaborate gamified activities with sustainable effects over time, it is fundamental to integrate elements that foster students’ intrinsic motivation. ^71^ ^,^ ^72^ In this regard, Turan et al. ^73^ highlight the importance of designing gamified experiences that not only use rewards but also promote meaningful changes in educational processes.

A crucial distinction to clarify is the difference between deep gamification and AI-driven personalization. While these two concepts are frequently addressed in conjunction, they embody fundamentally distinct constructs. Deep gamification involves the incorporation of significant and engaging game elements, including narrative, missions, collaboration, exploration, and formative feedback, which alter the fundamental framework of a learning activity to enhance intrinsic motivation, autonomy, competence, and relatedness. ^65^ ^,^ ^66^ The objective is to alter the way in which learners create meaning and interact with knowledge.

According to ^74^, deeps gamification promotes students` agency by encouraging exploration, meaningful choices, and risk management. Through these mechanisms, students become active participants in their learning process, which contributes to more sustained engagement.

Conversely, AI-driven personalization refers to a data-driven adaptive mechanism in which artificial intelligence algorithms tailor content, difficulty, sequencing, and feedback to each student’s cognitive profile, behavioral patterns, preferences, and emotional state. ^37^ ^,^ ^51^ ^,^ ^52^ Whereas deep gamification is grounded in pedagogical intentionality and narrative coherence, AI-driven personalization focuses on optimization and adaptation through continuous analysis of students’ data.

**Figure 2.**
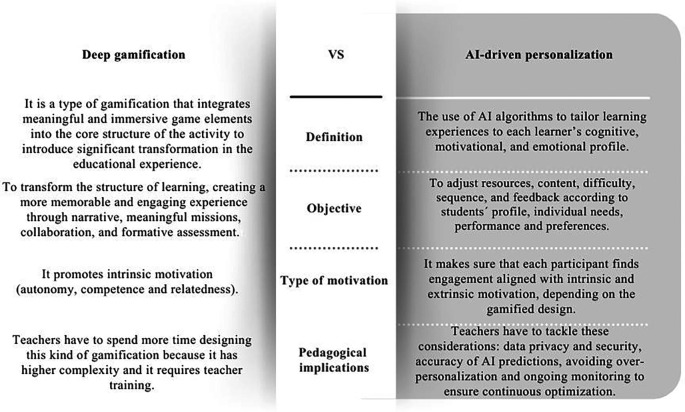
Comparative table on the differences between deep gamification and AI-driven personalization. Note: This figure presents the differences between Deep gamification and AI- driven personalization. Own creation based on Refs. 40, 47, 51, 52, 65, 66, 69, 70.

Understanding this distinction is essential to avoid confusing the pedagogical depth of gamification with the technical capacities of AI. As illustrated in Figure 2, which summarizes these conceptual differences and outlines their pedagogical implications, deep gamification may transform learning through meaning, narrative, and intrinsic motivation, whereas AI-driven personalization enhances adaptability and individualized support.

In this context, recent research has explored gamification and identified both advantages and challenges associated with its implementation. For example, Dah et al. ^75^warn that one of the main challenges of gamification is the prevalence of the “triad of badges, points, and leaderboards,” which fosters only extrinsic motivation and superficial engagement. While shallow gamification can spark interest through playful elements such as points and rewards, it does not significantly transform the learning experience. ^63^ ^,^ ^76^ ^,^ ^77^Its effects are often ephemeral ^66^ ^,^ ^77^ and tend to diminish in effectiveness when students lose interest in rewards, progressively reducing the effectiveness of these stimuli. ^79^ ^–^ ^81^Given these limitations, it is necessary to rethink gamification in the 21st century by incorporating elements that enhance intrinsic motivation and foster meaningful transformations. ^71^ ^–^ ^73^ Some studies emphasize that deep gamification can generate meaningful pedagogical changes in teaching–learning processes by integrating game mechanics into the core structure of the activities and creating narrative and immersive experiences that increase intrinsic motivation. ^78^

According to, ^40^ AI can strengthen the design of deep gamification experiences by generating personalized learning, providing immediate feedback, and creating more engaging interactive environments. From this perspective, artificial intelligence becomes a tool that allows teachers to redesign gamified experiences with greater pedagogical depth.

In this context, artificial intelligence can function as a resource that supports the development of deep gamification designs by helping teachers design gamified environments in a dynamic, adaptive, and student-centered way, offering personalized learning, immediate feedback, and more attractive interactive experiences. ^40^ However, the implementation of this type of gamification faces challenges related to time, pedagogical planning, alignment with learning objectives, and scalability. ^65^

After establishing the relevance of integrating artificial intelligence into gamification designs, explaining how this combination may foster 21st-century skills, and highlighting the need for deep gamification experiences supported by AI, this opinion article argues that the integration of artificial intelligence into gamification processes has the potential to support the design of deep gamification experiences by enabling highly personalized learning, aligned with students’ pace, styles, and needs, while also facilitating immediate feedback and more engaging interactive environments.

To clarify the current state of research on the integration of artificial intelligence and gamification, Table 1 presents a classification of the available evidence, distinguishing between empirically supported findings, emerging evidence derived from review-based studies, and conceptual propositions, thereby offering a structured overview of how knowledge in this field is being constructed. In addition to categorizing the literature, the table synthesizes the main characteristics, contributions, and reported limitations of each study. This classification not only situates the claim presented above within the research field, but also allows for a more critical understanding of the scope, strengths, and gaps in this emerging field where further research is needed.

**Table 1.** Table about types of evidence on AI-enhanced gamification: Empirical findings, emerging evidence and conceptual propositions.

Study	Type of evidence	Characteristics	Contribution to the Field	Reported Limitations
Liu ^52^	Empirical	Mixed-methods, quasi-experimental study with 486 undergraduate EFL students comparing adaptive learning paths, conversational agents, and interactive storytelling.	It provides comparative evidence that AI-powered gamification, especially adaptive learning paths, improves language proficiency and dynamic motivation.	Authors note a quasi-experimental design, reliance on self-reported motivation measures, and a 15-session intervention focused on short-term effects.
Laverde-Albarracín et al. ^82^	Empirical	Quantitative descriptive-correlational study with 70 secondary students; pre and post assessment with expert-validated test.	It shows a significant positive association between AI plus gamification and logical-mathematical performance, including a 40% increase in solving complex problems	No explicit study limitations were reported in the article text reviewed. The sample was small and it was intentional
Gao et al. ^79^	Empirical	Two-month quasi-experimental feasibility study with 456 university students using the *ShouTi Fitness* app.	It provides early evidence that AI-powered gamification can support engagement, usability, and adherence in physical activity interventions.	Authors report that physical activity frequency declined after 35 days; the 2-month period was insufficient for long-term sustainability assessment and there was no control group.
Bachiri et al. ^38^	Empirical	Study with 100 fifth-grade students in a MOOC Moodle environment using gamified automatic question generation for multiple-choice assessment.	It shows that gamified AI-supported assessment can improve student engagement, motivation, knowledge retention, and learning outcomes compared with conventional assessment.	Authors state that the sample was limited to fifth-grade students and the research lasted 3 weeks, so there is limited generalizability
Méndez Cabrera & Fajkišová ^83^	Empirical	Pilot action-research study in a public primary school using observation diaries, the implemented project, and meetings with the class tutor.	It offers evidence that AI-generated images, texts, and music can function as inclusive resources within gamified literary education, increasing participation and motivation.	The article frames the work as a pilot study and stresses the need for ethical use of AI in educational contexts.
Cabrera Félix & Román Santana ^84^	Emerging evidence (systematic review)	Qualitative systematic review of 15 articles (2021–2025) using PRISMA in Latindex, Dialnet, SciELO, and Scopus databases.	It synthesizes current trends and highlights that gamification, and AI can support motivating, personalized, and efficient learning while fostering critical skills and knowledge retention.	The review identifies implementation challenges related to teacher training, student adaptation, and ethical considerations.
Kumar (2022) ^39^	Empirical	Design and evaluation of a virtual-reality physical-training platform; includes experimental results and a survey with 30 participants.	It reports that artificial intelligence and virtual reality-supported gamified training improved learning performance and engagement in physical training.	The study had a small sample size and used self-reported data (perceptions).
Mohammed & Jesudas ^85^	Conceptual proposition	Conceptual discussion of AI and gamification in language learning, organized around personalization, conversational AI, evaluation, virtual reality and augmented reality and feedback mechanisms.	It clarifies how AI-supported personalization and gamified incentives may work together to enhance motivation, adaptive feedback, and immersive language practice.	The article explicitly notes constraints including accessibility, substantial resource requirements, and the risk of superficial engagement.
Pardim et al. ^86^	Conceptual proposition	This reflection article discusses educational and corporate transformations produced by the intersection of gamification and AI.	It contributes a human-centered conceptual lens, emphasizing personalization, adaptive feedback, inclusive design, and the role of gamified intelligent tutoring systems.	The paper highlights ethical and social risks, including excessive monitoring, manipulation of intrinsic motivation, and over-instrumentalizing learning
Ng ^87^	Emerging evidence (systematic review)	Systematic review of recent studies (from 2024 onward) on AI-powered gamification, with narrative synthesis of cognitive, emotional, and implementation themes.	It broadens the field by connecting AI-powered gamification not only to engagement and cognition but also to emotional intelligence and future-work readiness.	The review highlights barriers such as inadequate technological infrastructure, insufficient teacher training, cultural resistance, data privacy, and algorithmic bias.
Yuan ^88^	Emerging evidence (systematic review)	Systematic literature review of 28 studies on AI and gamification in university finance trading labs.	It shows how adaptive AI and gamification together can enhance engagement, knowledge retention, resilience, and decision-making agility in simulated trading environments.	The review identifies algorithmic bias, resource disparities, and ethical concerns as major constraints on equitable implementation. It included a small sample of studies, and it focuses on university finance trading simulations, so this limits generalizability.
Gómez Niño et al. ^40^	Emerging evidence (systematic review)	Systematic review following PRISMA with 175 articles and bibliometric visualization focused on AI, gamification, and 21st-century skills.	It shows that gamification and AI can jointly foster creativity, collaboration, communication, and critical thinking through personalized and socially interactive learning environments.	The article notes design challenges for teachers and warns that poorly implemented gamification may distract from learning. It only used a database.
Abbes et al. ^51^	Emerging evidence (review article)	Literature review on generative AI and gamification for personalized learning, covering generative artificial intelligence, adaptive learning, and adaptive gamification.	It systematizes how generative AI can support content creation and adaptive gamification for personalized learning experiences.	Authors emphasize that the integration of AI and gamification requires further research and highlight the need for adaptative models.
Bennani et al. ^37^	Emerging evidence (literature review)	Literature review on adaptive gamification in e-learning, synthesizing studies on user-centered design, player profiles, game elements, and AI integration.	It clarifies why adaptive, AI-enhanced gamification is needed to personalize learning based on learner profiles, interaction, and motivation.	Authors report limited application of adaptive gamification and AI in e-learning and lack of a general design framework.

Source Own elaboration. Note: This figure differentiates empirical findings, emerging evidence and conceptual propositions about the studies on AI-enhanced gamification

As shown in Table 1, although empirical studies report promising results, much of the current knowledge remains based on emerging and conceptual evidence, highlighting the need for more robust and long-term empirical research.

The following sections present conceptual arguments and selected research evidence that illustrate and support the position of this article from a theoretical perspective rather than through direct empirical demonstration. This approach is adopted because the integration of artificial intelligence into deep gamification remains an emerging field of discussion. As Artykbayeva et al., ^89^ argue, existing research mainly explores artificial intelligence and gamification in isolation; therefore, no definitive academic consensus has been established. Accordingly, this article provides a perspective contributing to an ongoing scholarly discussion.

## AI and deep gamification: A combination for personalized learning

After discussing the importance of developing deep gamified settings, it is necessary to examine more closely how the combination of deep gamification with artificial intelligence could create individualized experiences that fit each student’s needs, pace, and learning style.

Since educational systems must focus on the learner, personalizing earning is no longer simply a pedagogical goal but an urgent necessity. It is worth noting that, according to, ^51,^ artificial intelligence, when integrated into deep gamification designs, may support the development of learning environments tailored to the s the specific characteristics of each student. Complementary evidence from applied contexts ^36^ further indicates that the combination of AI and gamification can contribute to more personalized and engaging learning experiences. From a theoretical perspective, adaptative gamification models further propose that tailoring game elements to students` characteristics may contribute to the development of more interactive and motivating learning environments. ^47^

Conceptual and review-based studies on the integration of artificial intelligence and gamification suggest that this combination may support the personalization of learning experiences by adapting challenges, rewards, and feedback to students’ performance, preferences, and learning pace. ^51^ ^,^ ^90^ From a theoretical perspective, adaptive gamification models further propose that tailoring game elements to students`characteristics such as challenges, rewards and feedback may contribute to the development of more interactive and motivating learning environments. ^47^ ^,^ ^51^ ^,^ ^90^

Empirical and review-based studies such as that of Liu ^52^; Mohammed & Jesudas ^85^; Abbes et al. ^52^ and Bennani et al. ^37^ provide converging evidence suggesting that AI-driven gamification may support the customization of learning pathways and feedback mechanisms according to students' individual profiles, needs, and performance, although the strength and nature of this evidence vary across contexts and methodological approaches. In particular, Pardim et al., ^86^ highlight that the synergy between AI and gamification may support the personalization by leveraging real- time data analysis to identify students`needs and dynamically adapt gamified elements, such as task difficulty, feedback, and rewards. This conceptual study emphasizes the role of real -time data analysis in tailoring learning environments to individual learner needs. Additional support for this benefit is found in the systematic review by Cabrera & Santana, ^84^ whose analysis of 15 studies suggests that the integration of AI and gamification enhances motivating, personalized and efficient learning.

It is also essential to consider students’ diverse learning styles when designing AI-supported gamified environments Artificial intelligence can support the identification of students`learning styles and enable the development of personalized adaptative learning experiences. ^91^ Furthermore, aligning challenges and learning activities with students`needs and preferences may foster engagement and active participation. ^92^ ^,^ ^93^ In this sense, AI powered gamification has the potential to contribute to the design of personalized learning environments that support meaningful and engaging learning experiences. ^94^

These findings converge with evidence from systematic reviews, Ng ^87^ suggests that AI-powered gamification can enhance personalized learning, cognitive retention, and emotional intelligence through adaptive feedback, immersive environments, and data-driven learning pathways Additionally, Yuan ^88^ found that AI-driven personalization can tailor challenges to individual proficiency levels, suggesting that such adaptative mechanisms may contribute to improved academic outcomes.

Collectively, these studies, including Vélez White et al., ^95^ suggest that the integration of artificial intelligence into gamified designs has the potential to enable more dynamic and responsive interactions between students and their educational environments, thereby contributing to more individualized learning experiences. However, these propositions remain largely theoretical and require further empirical investigation.

From a conceptual perspective the integration of gamified designs and artificial intelligence may support the development of interactive learning environments in which students are actively involved in their learning processes. Gamified designs may encourage exploration, decision-making, and problem-solving in playful contexts, while AI may enhance these experiences through personalization, adaptive feedback, and real-time analysis. In this way, gamification and AI have the potential to foster students` autonomy, although evidence regarding their impact on meaningful learning and academic performance remains mixed and context- dependent. ^52^ ^,^ ^67^

## AI and deep gamification: An innovative alliance with real impact on learning

As noted in the previous section, the application of AI in education not only has the potential to personalize learning within gamified designs but also opens new possibilities for the development of teaching resources, the creation of interactive learning environments, and the enhancement of teaching and learning processes. ^51^

From a conceptual and empirical perspective, the literature suggests several emerging implications of integrating AI into gamification. These contributions can be grouped into three categories.

The first thematic category groups studies focused on engagement and motivation in AI-supportive learning environments. Evidence consistently suggests that AI-powered gamification strengthens students`motivation and engagement through personalization, adaptative feedback and competitive elements. Empirical studies such as Bachiri et al. ^38^, Liu ^52^ and Gao et al. ^79^ suggest that AI-enhanced gamification may contribute to increased learner motivation, participation, an engagement through personalized recommendations, competitive elements, and adaptive learning pathways.

The study by Bachiri et al. ^38^ aimed to examine whether AI-powered gamified assessments could enhance students’ engagement, motivation and learning outcomes compared to traditional approaches. Specifically, 92% of students reported that gamified assessments were engaging and enjoyable in comparison to 48% in traditional assessments, while 89% reported higher motivation compared to 54% in the non-gamified experience. This suggests that AI- powered gamification increases students’ interest and participation.

The study by Liu ^52^ compared three AI- enhanced gamification strategies: adaptive learning paths, conversational agents, and interactive storytelling. The results indicate that the combination of gamification and AI increased motivation, personalized the learning experience, and enriched learning, particularly through interactive storytelling that incorporated diverse cultural contexts.

Specifically, students valued the immediate feedback from conversational agents, the cultural richness of interactive storytelling, and the personalization of adaptive learning paths. Gamification elements such as leaderboards, unlockable content, and individualized challenges sustained interest and reinforced intrinsic motivation, contributing to improvements in both language proficiency and student engagement. In contrast, the control group, which followed a conventional course without AI or gamification, showed limited progress, suggesting that the integration of AI and gamification can enhance learning experiences by making them more meaningful, motivating, and personalized.

Although the study by Liu ^52^ is not explicitly framed within a critical pedagogy perspective, the inclusion of cultural elements into interactive storytelling suggests an effort to embed language learning into meaningful and contextually rich environments. This feature.can be interpreted as an attempt to link learning to meaningful contexts for students. This aspect aligns, at least partially, with the arguments of Teräs, ^6^ Williamson, ^7^ Facer, ^8^ who emphasize the importance of addressing the realities and needs of local communities.

The study by Gao et al. ^79^ aimed to evaluate the feasibility and usability of an AI-powered gamification intervention, implemented through the *ShouTi Fitness* app designed to enhance physical activity among college students. The findings indicate that the participants reported high levels of motivation and engagement associated with the use of AI-powered gamification features, including personalized recommendations, action recognition and dynamic adaptation,

Furthermore, the systematic review by Yuan, ^88^ that analyzed 28 studies in higher education, found that the integration of AI and gamification significantly improves engagement, knowledge retention, and decision-making in simulated learning environments. Moreover, review-based evidence, such as that provided by Ng, ^87^ highlights the role of adaptive feedback and personalized learning pathways in fostering students` motivation and engagement within AI-supported gamified environments.

Taken together, these findings suggest that the combination of AI-supported features and game-based elements may contribute to enhance students`engagement. However, current evidence remains largely short-term and context-dependent, which limits the conclusions that can be drawn regarding their long-term sustainability.

The second thematic category addresses learning outcomes and academic performance. Empirical and review-based studies provide converging, though uneven, evidence regarding the educational impact of AI-enhanced gamification on students ‘learning outcomes. Experimental findings, such as those reported by Laverde-Albarracín et al. ^82^ demonstrate significant improvements in problem-solving abilities and overall learning performance.

The literature review by Bennani et al. ^37^ provides a conceptual synthesis of adaptative gamification in e-learning, suggesting that incorporation of game elements and interactive components into immersive educational environments may enhance academic performance.

Moreover, the study by Gómez Niño et al ^40^ highlights the potential contribution of AI-enhanced gamification designs to the development of 21st-century skills, particularly critical thinking and problem-solving, based on a systematic review of the literature.

In contrast, studies such as Kumar ^39^provide more limited support, relying mainly on small-scale and perception-based data to suggest improvements in training effectiveness, without directly measuring cognitive outcomes or long-term retention. Taken together, these findings indicate that the potential of combining AI and gamification extends beyond motivational effects and may support learning outcomes. Nevertheless, the strength and generalizability of this evidence remain variable, depending on methodological rigor, context, and the type of outcomes assessed

These studies highlight the potential of AI-powered gamification to promote more student-centered collaborative learning processes through personalization and game-based interaction. However, the available evidence remains largely grounded in conceptual frameworks and context-specific applications, with limited empirical validation regarding its impact on meaningful learning and academic performance outcomes.

The third thematic category encompasses research examining AI as a support tool for teachers in gamified and data-driven learning contexts. Studies such as that of Vélez White, ^95^ Celik et al., ^97^ and Torres-Toukoumidis et al. ^98^ indicates that artificial intelligence may enhance educators`capacity to monitor students` progress, analyze learning- related data, and identify patterns associated with performance and engagement, thereby informing more targeted and contextualized instructional decisions. In particular, data driven systems and predictive models may support the identification of learning challenges and enable more timely data- informed interventions.

Beyond monitoring and data analysis, artificial intelligence may support teachers by efficiently filtering and organizing large volumes of information, thereby facilitating the pedagogical adjustments and enabling the adaptation of teaching methods and resources to students’ individual characteristics. ^95^ In this way, AI-supported gamified environments may contribute to more enhanced feedback mechanisms. Furthermore, Markauskaite et al. ^99^ highlight that AI-driven systems may support teaching processes by providing scalable feedback and learning analytics, helping teachers manage instructional tasks more effectively

However, these possibilities should be carried out critically, Giannakos et al. ^21^ emphasize that AI- generated feedback should be carefully evaluated and complemented by teachers own professional expertise. This caution is related to a broader consideration as noted by, ^57^ the effectiveness of these systems depends on teachers’ pedagogical decisions and their alignments with clearly defined instructional objectives.

### AI in action: Crafting a deep gamified experience

**Figure 3.**
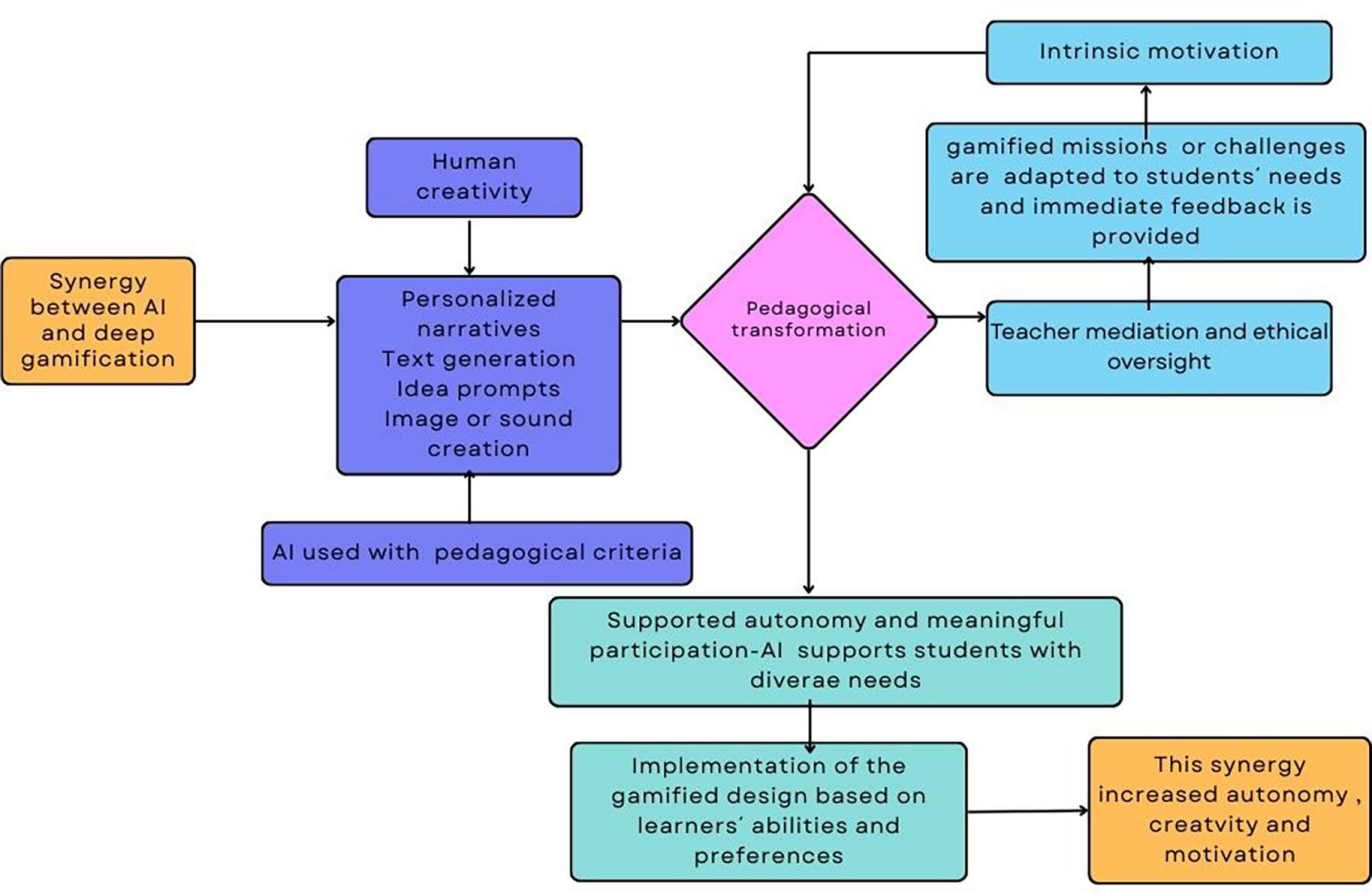
Diagram about how AI supports deep gamification.

This subsection presents an empirical example that shows how artificial intelligence can effectively support deep gamification in authentic educational settings. This example, complemented by Figure 3, shows how AI contributes to narrative enrichment, adaptive challenge design, multimodal resource creation, and inclusive participation, elements that characterize deep gamification and reflect the pedagogical principles discussed in earlier sections.

Own elaboration based on Mèndez Cabrera and Fajkisovà ^83^ study.

The study by ^83^ illustrates how AI can meaningfully support deep gamification in l specific educational contexts. In their study, AI enabled the personalization of narrative elements, contributed to content creation, provided immediate feedback, and scaffolded the writing process. AI tools adapted outputs to each student’s linguistic level, creativity, and personal preferences, making the experience both inclusive and authentic. Within a shared science-fiction narrative, students collaboratively developed characters, plots, and multimodal artefacts such as texts, images, and sounds, using generative AI tools including ChatGPT, Bing Image Creator, Leonardo, and Donna. The gamified experience integrated narrative missions, teamwork, and formative feedback, allowing each student to co-create within a common story world. It’s worthy to highlight that the design fostered inclusion by enabling students with diverse abilities and learning needs to participate actively and express themselves through modalities aligned with their skills and interests. Throughout the process, teachers guided the process, ensuring ethical AI use, and maintaining a clear focus on pedagogical goals rather than technological novelty.

This project exemplifies a deep gamification design because it incorporates game elements such as narrative immersion, adaptive missions, and collaboration within a meaningful, student-centered pedagogical framework. Instead of relying on external rewards, the gamified system fostered intrinsic motivation, autonomy, and reflection by transforming learning into a creative quest. Artificial intelligence was integrated into this design and it allowed to make the experience both inclusive and authentic. The synergy between AI and deep gamification therefore supported autonomous, meaningful, and multimodal learning, illustrating how technology can enrich education when guided by sound pedagogical principles (see Figure 3).

In summary, the combination of deep gamification and artificial intelligence entails significant implications for teaching practice and the optimization of teaching and learning processes at all educational levels. In this sense, teachers should rely on AI to design gamified environments that respond to students’ interests and needs. ^53^ This requires moving toward deeper pedagogical proposals that integrate meaningful narratives, adaptive feedback, and contextualized challenges, overcoming mechanical models focused exclusively on extrinsic rewards. ^100^ ^,^ ^101^

Similarly, it is imperative that students engage actively in their educational journey, fostering the growth of autonomy and decision-making abilities. Strategies like creating personalized avatars or customizing aspects of the gamified environment may enhance identification, boost engagement, and reinforce commitment to learning, so promoting intrinsic motivation.

The utilization of gamification analytics technologies, such as GameAnalytics, in AI-enhanced gamified environments enables educators to more precisely track student interactions with gamified systems. By visualizing and analyzing learning data produced by AI, such as speech, gestures, and student action logs, educators may receive feedback that can help them adjust game dynamics and refine gamification designs, which may contribute to increased engagement, motivation, and potentially improved learning outcomes. ^21^ ^,^ ^102^ This process may foster a form of co-creation between educators and artificial intelligence, designed that has the potential to enhance the educational experience and substantially improve teaching and learning methodologies.

## A critical reading of current evidence: Point of agreement, gaps and open questions

A thematic analysis of the literature reveals a growing, though still partial, consensus regarding the educational potential of integrating artificial intelligence and gamification. Empirical, applied, and review-based studies consistently associate this combination with improvements in student motivation, engagement, and the personalization of learning experiences. Empirical studies such as those by Liu ^52^ provide empirical evidence that AI-enhanced gamification significantly improves language proficiency and motivation in EFL contexts. Similarly, the empirical study by Bachiri et al. ^38^ demonstrate that gamified assessments using AI-driven automatic question generation increase student engagement and improve learning outcomes. The systematic review by Ng ^87^ and the emerging evidence study by Abbes et al. ^51^ also highlight the potential of this synergy for adaptive feedback and more student-centered learning, reporting positive evidence. In addition, systematic reviews such as Gómez Niño et al. ^40^ argue that the synergy between gamification and AI fosters 21st-century skills, including problem-solving and critical thinking, while Yuan ^88^ suggests that AI-supported gamification may contribute to adaptive feedback mechanisms, knowledge retention, and more student-centered learning processes.

However, it is important to note that much of this evidence remains context-dependent and uneven in terms of methodological strength. For example, Bachiri et al. ^38^ point out limitations such as convenience sampling and a focus on single grade levels. Gao et al. ^78^ used a quasi-experimental design without a control group; therefore, causal findings could not be determined. These limitations restrict the strength of the conclusions that can be drawn at this stage.

At the same time, several important areas of ongoing debate emerge and deserve careful consideration. First, the long-term effectiveness and generalizability of AI-enhanced gamification designs remain uncertain, as many studies rely on short-term interventions, pilot implementations, or samples drawn from specific disciplines. This is illustrated, for instance, by Gao et al. ^78^, whose findings are based on a brief intervention with a specific population.

Consequently, many authors emphasize the urgent need for longitudinal studies and larger, more diverse samples to ensure applicability across different cultural and socioeconomic contexts. Second, although several authors suggest that combining AI and gamification may produce benefits, empirical evidence directly indicates that this combination is more effective than using either approach independently and remains limited and inconclusive. Third, there is no clear consensus regarding which specific design elements are most effective across educational contexts, as outcomes vary depending on student profiles, subject areas, and implementation strategies. Fourth, significant gaps remain in the current understanding of this synergy. These include demographic imbalances, as most research is concentrated in higher education and STEM disciplines, as well as a lack of attention to socio-affective factors, particularly the socio-emotional dimensions of AI-gamified experiences. Finally, recent literature consistently highlights ethical concerns related to algorithmic bias, data privacy, and unequal access to technology. This suggests that the pedagogical promise of these systems must be considered alongside their limitations, risks, and the conditions required for their responsible implementation.

## Beyond optimistic perspectives. Rethinking the Effectiveness of Gamification and AI in Learning

There are divergent findings and emerging debates in gamification and AI research. The empirical findings on gamification are not always consistent. For instance, the results of the empirical study by Bueno-Baquero ^103^ which analyzed the integration of gamification for promotion and training in PC with 99 future teachers, using a control group with which a superficial gamification design was implemented and an experimental group with which deep gamification was used, revealed mixed outcomes regarding the effectiveness of deep gamification compared to shallow gamification. While pre-service teachers exposed to the deep gamified environment reported improvements in certain dimensions of self-perceived computational thinking skills, contrary to expectations, the control group showed a decrease in the problem-solving dimension after the implementation of the gamified PC instruction.

Moreover, the results showed that the control group, which was exposed to shallow gamification, obtained higher scores in intrinsic motivation, whereas the experimental group, which was exposed to deep gamification, had lower scores in intrinsic motivation and also higher amotivation. These findings are striking because they contrast with results reported in the literature by authors such as Sánchez Nolasco, ^74^ Mozelius et al. ^66^ and Gurjanow et al. ^65^ and who have argued an association between intrinsic motivation and deep gamification and, on the other hand, between extrinsic motivation and superficial gamification.

Additional evidence also suggests that there is a need for further investigation in relation to the long-term effects of gamification. The empirical study by Correa Rufino et al., ^104^ which examined the integration of gamification and chatbots in educational environments through a mixed-methods approach that combined AI-based simulations and real-world field research, found that while gamified systems increased user interaction and engagement, which was demonstrated through increases in interaction time and click rates, the results did not show a clear relationship between these metrics and improved knowledge retention, so the link between gamification and enhanced learning outcomes remains inconclusive.

Moreover, this research concludes that although gamification may enhance participation and attention, it is necessary to examine its long-term impact on learning outcomes and its ability to support knowledge construction in different educational contexts. Also, it highlights that the successful implementation of gamified approaches depends on adequate technological infrastructure, teacher training, and institutional governance.

Regarding limitations and challenges, the conceptual study by Li et al., ^105^ suggests that although the integration of artificial intelligence into gamified learning environments may enhance students’ motivation, engagement, and performance, several implementation challenges may complicate these outcomes. In the context of this study, the use of complex AI algorithms and multimedia-rich gamified content was associated with technical drawbacks, including system glitches and platform incompatibilities when AI-driven gamification is integrated with existing educational technologies. From a conceptual perspective such difficulties may also involve the need for frequent systems updates and adaptations to maintain compatibility with new software versions, hardware devices, and web browsers. Neglecting such updates may lead to reduced system performance and functionality, potentially affecting students’ motivation.

Although, the empirical study by Gao et al. ^79^ reported an initial increase in participation activity levels declined after approximately 35 days. Also, a minority of participants, around 10%, indicated that some AI-generated exercise reported that approximately 10% of participants perceived some AI-generated exercise recommendations were repetitive or insufficient tailored to their fitness levels, highlighting the need for further refinement of personalization algorithms. Moreover, Hallifax et al. ^58^ caution that modifying game aspects to correspond with students’ psychological profiles presents an ethical quandary: personalized gamification may exploit learners and curtail their agency. In the same way, too much adaptation might make pupils only interact with things they are already comfortable with, which can slow their growth and make it harder for them to deal with new problems.

Taken together, the findings discussed in this section suggest that the relationship between AI-enhanced gamification and learning outcomes is complex due to the inconsistent motivational results, limited evidence linking engagement metrics to meaningful learning, technical implementation challenges, and concerns about student`s agency indicate that the potential of this combination cannot be taken for granted.These limitations are not only practical in nature; they also point to deeper questions regarding the ethical and technological conditions under which AI-optimized gamification operates. Addressing these questions requires moving beyond performance-focused perspectives and examining more critically the values and assumptions embedded in these systems, which is precisely the focus of the following subsection.

## Between potential and the gap: Ethical and technological challenges of AI-optimized gamification

Despite the pedagogical potential of integrating artificial intelligence with deep gamification, its implementation also poses a number of important challenges. While the previous section discussed practical and implementation-related limitations, additional ethical and technological concerns also deserve attention. This section examines other important challenges that teachers have to deal with.

The first challenge concerns data protection, ethics, and privacy. Gamification mediated by artificial intelligence collects large volumes of information about students, including their academic progress, in-game behavior, preferences, and even demographic data, which poses risks if the security and confidentiality of such records are not guaranteed. ^106^ ^,^ ^107^ To address this issue, it is essential to establish strong security measures such as data protection protocols and audits of AI models to detect bias and prevent misuse. ^38^ ^,^ ^40^

The second challenge has to do with avoiding excessive dependence on technology. AI should be understood as a means and not an end in educational processes. ^95^ Therefore, it is not advisable to fully delegate the design of gamified experiences to artificial intelligence, especially regarding content personalization, the difficulty of challenges and narratives, since game mechanics must always respond to pedagogical objectives. ^40^ ^,^ ^108^ In this sense, AI should play a complementary role, contributing to analysis, monitoring, and optimization, while pedagogical design remains the responsibility of the teacher. ^109^

The integration of AI into deep gamification requires a pedagogically grounded approach that balances innovation with caution, ensuring that adaptive and generative technologies enrich the learning experience. Based on the ^59^ AI Competency Framework for Teachers, the fourth competence called *AI pedagogy integration* of the Deepen level provides a balanced model for reconciling AI’s potential with the risks of over-dependence because according to this competency teachers should skillfully integrate AI into designing and guiding student-centered learning to foster engagement, support individualized instruction, and improve teacher-student interactions with the purpose of promoting critical thinking, problem-solving skills, and empathy among students. This competency involves evaluating AI tools pedagogical affordance for student-centric pedagogical activities, in this case for gamified designs, cooperating with peers or experts to assess whether the design of generative AI systems takes into account pedagogical implications, designing student-centric teaching and learning activities based on validated educational AI tools and avoiding the use of AI automate the design, administration and grading of assessments by analyzing the risks of AI in usurping human responsibility when providing feedback and making decisions on students’ learning outcomes.

The third challenge relates to the technological and infrastructural gap. Significant inequalities persist in access to, use of, and proficiency with information, communication, and artificial intelligence technologies. ^68^ UNESCO, ^44^ in its 2023 agenda, stresses that young people must be guaranteed access to both formal and informal educational experiences that broadly integrate technology to ensure greater equity in opportunities.

This gap affects both teachers and students who lack technological resources in their educational institutions. ^,^ ^85^ ^,^ ^110^Neubaum et al. ^111^showed that during the pandemic, advances in digital skills mainly benefited young people with greater resources, thus deepening inequalities rather than reducing them. According to, ^95^this exclusion stems from social, demographic, and educational factors that limit equitable access to the benefits of technological advances.

With respect to infrastructure, the need to provide educational institutions with both physical and digital resources has become evident: devices, connectivity, educational software, digital platforms, and tools for developing AI-supported gamified experiences. ^110^ The lack of such resources restricts the effective implementation of new proposals, especially in developing countries where investment in technological equipment remains insufficient. ^95^ In this context limited internet connectivity and restricted access to high-quality devices may prevent some students from fully participating in AI-driven gamified learning environments. ^105^

The fourth challenge involves the cultural and pedagogical appropriation of technology. As Feenberg ^112^ argues, technologies are not neutral, but neither are they closed: they can be adapted and reconfigured according to context. However, as Watters ^113^warns, the problem does not lie in the rigidity of technology but in the uncritical adoption of external models, particularly from the Global North, without adapting them to local contexts, languages, and teaching practices. De Sousa Santos and Meneses ^114^ reinforce this idea, by pointing out that it is not about incorporating tools without reflection but about questioning their meaning within situated educational processes. Instead of replicating foreign uses, it is more relevant to start from our own educational and cultural needs. Innovation, in this sense, should be conceived as situated creation rather than mere imitation. Furthermore, culturally inappropriate content in AI-enhanced gamified designs may lead to student disengagement, that’s why it is essential to design culturally inclusive learning materials. ^105^

The fifth challenge relates to teacher training, a fundamental aspect for harnessing the potential of AI-mediated deep gamification. According to ^51^ the lack of specialized preparation significantly limits its impact in education. Dah et al. ^75^agree that the effectiveness of gamification depends on multiple factors: design quality, theoretical grounding, ^116^ ^,^ ^117^standardization of elements, ^116^individual differences, ^118^ and contextual particularities. ^119^ These conditions underscore the need for teachers trained in gamification software, ^120^ capable of adapting resources to their educational contexts and to the characteristics of their students. ^121^

In order to progress toward meaningful implementation, it is imperative to provide training to both in-service and preservice teachers on the development of gamified experiences that incorporate AI as a support tool. This training should encompass the acquisition of critical digital literacy skills, the comprehension of adaptive systems, and the mastery of resources such as immersive narratives, dynamic feedback, and contextualized challenges, the creation of meaningful rewards, the integration of AI-driven gamification into the existing curriculum, and the effective management of classroom dynamics in gamified environments. ^105^

Finally, despite the challenges, the future of AI-supported gamification. may hold great potential This technological convergence may convert learning into a more dynamic and motivating experience by encouraging student participation and allowing the continuous adaptation of gamification designs throughout the learning process. ^102^ IIn this context, addressing current challenges from a critical and proactive perspective will make it possible to fully leverage thel optimizing capacity of AI in deep gamification environments. Ultimately, true innovation, as Örpek et al. ^36^ argue, will depend on whether educational institutions and educators manage to balance the intelligent use of technology with situated pedagogical objectives, ensuring that educational practices respond to the needs of local communities.

## Conclusions

This opinion article has examined how the integration of artificial intelligence into deep gamification designs may support meaningful changes in learning environments, providing that such integration is guided by critical pedagogical principles and a clear understanding of both its potential and its current challenges

The analysis underscores the significance of acknowledging that artificial intelligence does not supplant the teacher’s function but rather augments it by providing resources for personalization, real-time feedback, and automated material development. The gamification of educational experiences relies on the educator, who, from a critical standpoint, crafts narratives and challenges while considering the reality, interests, needs, and learning styles of their pupils.

The literature analysis emphasizes the importance of identifying what is currently supported, what appears promising and what remains conditional or speculative in this field. On the one hand, there is consistent evidence drawn from empirical studies suggesting that the integration of AI and gamification can contribute to improvements in student motivation, engagement, and, in certain contexts, academic performance. However, these findings, while encouraging, are largely context-dependent, rely on short-term interventions, and in some cases present methodological limitations.

On the other hand, a substantial body of review-based and conceptual evidence suggests that the combination of AI and deep gamification has the potential to transform learning processes through personalization, adaptive feedback, and the development of 21st-century competencies. However, as these studies themselves acknowledge, this potential has not yet been consistently validated through robust empirical research, and real-world implementation examples remain limited. Furthermore, other findings underscore that the relationship between design complexity and learning outcomes remains an open and unresolved question.

In this context, several challenges must be explicitly recognized as priorities for ongoing research and practice. The long-term effectiveness of AI-enhanced gamification designs across diverse educational contexts remains insufficiently studied and constitutes an urgent research need. The absence of consensus regarding which design elements are most effective, the demographic imbalance in the available literature since most articles concentrate in higher education and STEM disciplines and the underexplored socio-affective dimensions of AI-gamified experiences represent relevant gaps that future research must address.

This analysis also makes clear that combining AI and deep gamification is not merely a technological upgrade but suggests a shift in how human and artificial intelligence can interact within educational environments, converting static gamification into dynamic, adaptable, and learner-centered educational experiences. ^37^. This requires teachers to design meaningful learning experiences instead of just giving students fun activities to do to keep them interested in class, ^122^ revise their pedagogical approaches facilitate the acquisition of 21st-century competencies, and develop the specific competencies outlined in the UNESCO AI Competency Framework for Teachers to critically evaluate algorithmic outputs, resolve ethical dilemmas, and make pedagogically sound decisions. In this sense, AI-enhanced deep gamification may offer promising opportunities for educational innovation, but only when anchored in a human-centered, pedagogically deliberate, and ethically accountable framework.

The potential of artificial intelligence and gamification to support meaningful educational change depends on a solid pedagogical foundation and proper implementation, which entails considering a set of critical boundary conditions and prerequisites that must be addressed. From a technological standpoint, a robust technological infrastructure is fundamental, as these systems depend on stable high-speed internet connectivity, advanced hardware, and secure data management platforms to function effectively. At the pedagogical level, teacher training emerges as an essential requirement; educators must develop not only specific digital competencies to manage AI-driven tools, but also the critical digital literacy to design meaningful gamified experiences that respond to the real needs of their students. Also, institutional governance and clear ethical frameworks are equally indispensable, as they provide the structures needed to oversee data privacy, mitigate algorithmic bias, and ensure that human-centered pedagogical principles remain at the core of the design process. Finally, and in alignment with the critical pedagogical perspective that informs this opinion article, contextual factors, including cultural adaptation, students' technological literacy, and the socio-economic realities of each learning environment, must be carefully considered to prevent the digital divide from widening and to ensure that AI-enhanced gamification designs are inclusive for all learners.

## F1000 AI policy

We have read and agree to comply with the F1000Research AI Policy. We confirm that, in accordance with this policy, ChatGPT-5 was used for style correction review. In addition, generative AI tools were employed in the initial design of the figures included in this article, which were subsequently refined, edited, and validated by the authors to ensure accuracy and alignment with the manuscript’s objectives. All uses of generative AI were conducted under the supervision of the authors, with full transparency and rigorous review.

## Data availability

No data are associated with this article.
