# Supplementary material for: Deep Gamification and Artificial Intelligence as Catalysts of Educational Transformation
Source: F1000Res. 2026 Jun 1;14:1156. Originally published 2025 Oct 22. [Version 4] doi: 10.12688/f1000research.171453.4 (PMC13161853; doi:10.12688/f1000research.171453.4)
Supplement: Peer review history

# Reviewer report: Reviewer response for version 4

**DOI:** 10.5256/f1000research.202117.r479874

**Published:** 2026-06-15

**Authors:** Nur Azaliah Abu Bakar

**Affiliations:** 1 Universiti Teknologi Malaysia, Skudai, Johor, Malaysia

The article discusses a timely and relevant issue: the integration of artificial intelligence and deep gamification in education, framed through critical pedagogy. It argues that AI can support personalised, adaptive and meaningful gamified learning when guided by teacher agency, ethical oversight and sound pedagogical design. The revised manuscript is stronger than earlier versions because it now clarifies its theoretical and integrative purpose, distinguishes deep gamification from AI-driven personalisation, includes a method section, presents an evidence classification table, and acknowledges mixed findings, ethical concerns, infrastructure limitations, teacher training needs and contextual constraints.

 The topic is generally discussed accurately in relation to current literature. The article engages with recent work on AI in education, gamification, adaptive learning, teacher competencies and critical pedagogy. The inclusion of UNESCO’s AI competency framework, systematic reviews and empirical examples improves the scholarly grounding. However, some parts still require refinement to avoid overgeneralisation. Claims about learning styles, improved academic outcomes and educational transformation should remain carefully qualified because the cited evidence is still context dependent, short term and uneven.

 Factual statements are mostly supported by citations, but not all are sufficiently precise. Some sections rely on broad claims supported by conceptual or review based literature rather than direct empirical evidence. The discussion of AI identifying learning styles, for example, should be framed cautiously because learning styles remain contested in educational research. The manuscript should also correct repeated text, typographical errors and citation inconsistencies, particularly in the duplicated explanation of the UNESCO “Acquire” level and several language issues across the manuscript.

 The arguments are coherent and relevant, but the evidentiary support remains partly uneven. The article provides useful empirical and conceptual examples, including Table 1, which separates empirical findings, emerging evidence and conceptual propositions. Nevertheless, the manuscript would be scientifically stronger if it more explicitly synthesised the strength, limits and contexts of the empirical studies instead of listing them descriptively. The authors should also clarify which claims are supported by empirical evidence, which are emerging from review based evidence, and which remain conceptual propositions.

 The conclusions are broadly aligned with the argument, but they remain somewhat aspirational. The manuscript appropriately recognises that AI enhanced deep gamification depends on teacher training, infrastructure, governance, ethical safeguards and contextual adaptation. However, the conclusion should further reduce transformative language and more explicitly state that long term effectiveness, scalability and impact on meaningful learning remain open empirical questions.

 Points that must be addressed for scientific soundness are as follows. First, moderate all remaining broad claims on transformation, learning outcomes and learning styles. Second, correct repetition, typographical errors and citation inconsistencies. Third, strengthen the synthesis of evidence by explaining the methodological limits of the cited studies more analytically. Fourth, ensure the conclusion clearly distinguishes established evidence from promising but unproven claims. With these revisions, the article would offer a balanced and useful conceptual contribution to the literature on AI, gamification and critical pedagogy.

Is the topic of the opinion article discussed accurately in the context of the current literature?

Yes

Are arguments sufficiently supported by evidence from the published literature?

Partly

Are all factual statements correct and adequately supported by citations?

Partly

Are the conclusions drawn balanced and justified on the basis of the presented arguments?

Partly

Reviewer Expertise:

Artificial Intelligence, Data Analytics, Enterprise Architecture, Digital Transformation, Information Systems Governance, Educational Technology, Cybersecurity, and Higher Education Internationalisation.

I confirm that I have read this submission and believe that I have an appropriate level of expertise to confirm that it is of an acceptable scientific standard, however I have significant reservations, as outlined above.

# Reviewer report: Reviewer response for version 4

**DOI:** 10.5256/f1000research.202117.r489887

**Published:** 2026-06-05

**Authors:** Rocsana Manea Tonis

**Affiliations:** 1 National University of Physical Education and Sports, Bucharest, Bucharest, Romania

 The manuscript discusses AI and gamification, but lacks a clearly defined central contribution, making it read more like a conceptual reflection than a framework paper. Critical engagement with issues such as algorithmic bias and data privacy is limited, with an overly optimistic tone. Claims made about AI's impact on learning lack robust evidence, making it appear less credible. The term "deep gamification" is not sufficiently operationalized; clearer definitions and measurements are necessary. Overall, while the paper is suited for publication as an opinion piece in F1000Research, it requires clearer definitions, stronger theoretical positioning, and deeper engagement with risks and limitations to enhance its scholarly impact.

**Recommendation: **I approve this article; however, I recommend that the authors address the above suggestions for further improvement.

Is the topic of the opinion article discussed accurately in the context of the current literature?

Yes

Are arguments sufficiently supported by evidence from the published literature?

Yes

Are all factual statements correct and adequately supported by citations?

Yes

Are the conclusions drawn balanced and justified on the basis of the presented arguments?

Yes

Reviewer Expertise:

AI driven solutions

I confirm that I have read this submission and believe that I have an appropriate level of expertise to confirm that it is of an acceptable scientific standard.

# Response

**Published:** 2026-06-05

**Authors:** Ana Dolores Vargas Sánchez

**Affiliations:** Cundinamarca, Universidad de La Sabana, Chia, Cundinamarca, Colombia

We thank the reviewer for their thoughtful and constructive feedback. We appreciate the opportunity to strengthen the manuscript and address the concerns raised.

# Reviewer report: Reviewer response for version 3

**DOI:** 10.5256/f1000research.198530.r478851

**Published:** 2026-05-11

**Authors:** Alexandhrea Hiedie Dumagay

**Affiliations:** 1 Western Mindanao State University, Zamboanga City, Philippines

The revised manuscript demonstrates clear and substantial improvement in response to the previous review comments. The authors have strengthened the conceptual positioning of the article and now more clearly frame the manuscript as a theoretical and integrative contribution rather than an empirical study. This clarification improves the alignment between the article’s aims, discussion, and conclusions.

 The manuscript also presents a more balanced discussion of the literature. The authors have moderated several previously strong claims and now distinguish more carefully between empirically supported findings, emerging evidence, and conceptual perspectives. The inclusion of the evidence classification table and the expanded discussion of mixed findings, implementation challenges, and contextual limitations considerably strengthen the scholarly grounding of the article.

 In addition, the discussion of teacher agency, ethical considerations, algorithmic bias, and infrastructural inequalities provides a thoughtful perspective on the integration of AI and deep gamification in educational settings. The manuscript now offers a more reflective and critically informed contribution to ongoing conversations in the field.

 The article is timely, relevant, and well connected to current scholarship. The revisions have improved the overall coherence and balance of the manuscript while preserving its original conceptual contribution. A few minor language and stylistic refinements could further improve readability and conciseness in some sections, although these do not substantially affect the overall quality of the work.

 Overall, this revised version represents a meaningful improvement and makes a valuable contribution to discussions on AI, gamification, and critical pedagogy in education.

Is the topic of the opinion article discussed accurately in the context of the current literature?

Yes

Are arguments sufficiently supported by evidence from the published literature?

Yes

Are all factual statements correct and adequately supported by citations?

Yes

Are the conclusions drawn balanced and justified on the basis of the presented arguments?

Yes

Reviewer Expertise:

Social Sciences, Education and technology, Artificial Intelligence in Education

I confirm that I have read this submission and believe that I have an appropriate level of expertise to confirm that it is of an acceptable scientific standard.

# Reviewer report: Reviewer response for version 2

**DOI:** 10.5256/f1000research.193431.r446667

**Published:** 2026-02-03

**Authors:** Alexandhrea Hiedie Dumagay

**Affiliations:** 1 Western Mindanao State University, Zamboanga City, Philippines

The opinion article addresses a timely and relevant topic by exploring the intersection of artificial intelligence, deep gamification, and critical pedagogy in contemporary education. It presents a clear conceptual framing in which pedagogy guides the use of gamification and AI, and it thoughtfully considers both potential benefits and challenges, including teacher agency, ethical issues, and contextual constraints. The manuscript demonstrates strong engagement with contemporary literature and raises important questions for the field, while some areas could benefit from further clarification to strengthen coherence.

 Overall, the topic is discussed appropriately in relation to current studies. The authors draw on a wide range of recent sources and align their discussion with ongoing debates about ethics, pedagogy, and technology in education. In a few places, however, the manuscript appears to go slightly beyond what the current evidence can firmly support, particularly when describing the transformative impact of AI-enhanced gamification. It may therefore be helpful to signal more clearly when claims are conceptual or aspirational rather than empirically established, and to engage more explicitly with studies that report mixed or critical findings.

 The manuscript is well referenced, and most claims are supported by appropriate citations. Some statements are framed in relatively strong terms compared to the context-specific nature of much of the existing evidence. Moderating the language of these claims and providing brief contextual details when referring to key studies would further strengthen the scholarly grounding.

 The conclusions are consistent with the overall argument and appropriately emphasize teacher agency and ethical awareness. They could be further strengthened by distinguishing more clearly between established findings, emerging evidence, and longer-term possibilities. Overall, this is a thoughtful and relevant contribution, and it has the potential to offer an even stronger and more balanced contribution to ongoing discussions in the field.

Is the topic of the opinion article discussed accurately in the context of the current literature?

Yes

Are arguments sufficiently supported by evidence from the published literature?

Yes

Are all factual statements correct and adequately supported by citations?

Yes

Are the conclusions drawn balanced and justified on the basis of the presented arguments?

Yes

Reviewer Expertise:

Social Sciences, Education and technology, Artificial Intelligence in Education

I confirm that I have read this submission and believe that I have an appropriate level of expertise to confirm that it is of an acceptable scientific standard, however I have significant reservations, as outlined above.

# Response

**Published:** 2026-04-06

**Authors:** Ana Dolores Vargas Sánchez

**Affiliations:** Cundinamarca, Universidad de La Sabana, Chia, Cundinamarca, Colombia

We appreciate the fourth reviewer's comments and ideas on the document. It was edited to stress the importance of being careful about what you say and to stay away from making statements that aren't backed up by enough evidence. The wording in the text was carefully toned down by replacing strong causal phrases with more cautious ones like "suggests," "indicates," and "has the potential to." It also changed normative statements by using modal auxiliaries like "may" and "could." A new introductory paragraph was added before the section "AI and deep gamification: A combination for personalized learning" to make it clear that the arguments made are only theoretical and that putting AI into deep gamification is a new field that hasn't yet reached a clear academic consensus.

 To improve the strength of the arguments and balance the evidence base, the literature was sorted into clear thematic groups, and new sections were added to bring together both similar findings and mixed or critical results. The section "Beyond optimistic perspectives: Rethinking the effectiveness of gamification and AI in learning" was added. It includes contextual evidence and counterarguments. The subsection "A critical reading of current evidence: points of agreement, gaps, and open questions" identifies areas of emerging agreement, such as motivation, engagement, and personalization, as well as open debates and methodological limitations that keep coming up. Table 1 also talks about the studies' limitations in a methodical way. It contains information about the methodological design, sample size, and disciplinary scope.

 Lastly, the findings were carefully changed so that they better reflect the current state of the data. They are now organized into three groups based on strength: conclusions that are already backed up by empirical investigations, promising but not yet strongly validated data, and questions that are still open and need more research. We took eliminated the speculative remarks and added a section that clearly lists the most important problems for future study, such as the lack of longitudinal studies, contextual inequities, and ethical considerations. A last paragraph was also included that talked about the things that need to be in place for responsible implementation, like the technology infrastructure, teacher training, institutional governance, and being aware of the educational setting.

# Reviewer report: Reviewer response for version 2

**DOI:** 10.5256/f1000research.193431.r444565

**Published:** 2026-01-13

**Authors:** Aleksandra Porjazoska Kujundziski

**Affiliations:** 1 International Balkan University, Skopje, Skopje, North Macedonia

**Summary of the article**: This article examines the intersection of artificial intelligence (AI) and deep gamification as a means to achieve educational transformation. Drawing on critical pedagogy, it is argued that AI should serve as a supportive and adaptive component within pedagogically intentional gamified frameworks, rather than acting as an independent catalyst for change. The article differentiates deep gamification from superficial, reward-based methods and from AI-driven personalization by emphasizing narrative, meaning-making, intrinsic motivation, and learner agency. Through a conceptual synthesis of recent literature and selected empirical examples, the authors identify synergies among pedagogy, gamification, and AI, highlighting benefits such as personalization, adaptive feedback, increased engagement, and the development of 21st-century skills. The article also addresses challenges such as algorithmic bias, ethical oversight, teacher autonomy, data privacy, infrastructural inequality, and the necessity for professional development. The conclusions assert that educational transformation can only occur when AI-enhanced gamification is directed by critical, human-centered pedagogy and responsible teacher involvement.

**Accuracy of topic discussion in relation to current literature:** The manuscript demonstrates strong integration with recent and relevant literature, especially post-2020 scholarship on AI in education, gamification, critical pedagogy, and teacher competencies. Its conceptual framework is consistent with prevailing contemporary discourses that prioritize ethical AI use, teacher agency, and pedagogical intentionality. Nevertheless, the manuscript offers limited engagement with alternative or dissenting perspectives. Although potential risks are acknowledged, the discussion predominantly highlights literature that supports the authors’ position. More skeptical viewpoints, including studies reporting mixed, null, or context-dependent effects of gamification and AI, receive insufficient attention.

 Recommendations: Contrasting findings from the literature, such as limited learning gains, motivation decay, and implementation failures, should be explicitly integrated. A concise subsection should synthesize critical or inconclusive evidence, clarifying areas of consensus and ongoing debate. The article’s stance should be clearly presented as one perspective within a continuing scholarly discussion, rather than as an emerging consensus.

**Factual accuracy and citation support: **Most factual statements are accurate and substantiated by appropriate citations, including peer-reviewed studies and authoritative frameworks (UNESCO, OECD). The literature base is comprehensive and generally well chosen. However, certain claims, particularly those characterizing impacts on learning outcomes as “significant,” “radical,” or “transformative,” are articulated in broad terms, whereas the supporting evidence is frequently context-specific, small-scale, or illustrative. Furthermore, some normative assertions are conveyed using language that closely resembles empirical claims.

 Recommendations: Strengthen the alignment between claims and evidence by replacing strong causal language with qualified phrasing such as “suggests,” “indicates,” or “has potential to.” Clearly specify when claims are conceptual or normative rather than empirically established. Where feasible, incorporate meta-analyses, systematic reviews, or large-scale studies to substantiate general claims regarding effectiveness. Additionally, consider including a table or box that differentiates empirically supported findings, emerging evidence, and conceptual propositions.

**Strength of evidence supporting the arguments: **The arguments presented are coherent and logically developed, with consistent connections to published literature. The manuscript effectively integrates conceptual reasoning with selected empirical examples. However, the evidentiary support remains uneven. Numerous references serve illustrative purposes rather than building a cumulative case, and counter-evidence is not examined in detail. Consequently, certain arguments appear more persuasive rhetorically than they are substantiated empirically.

 Recommendations: Enhance argumentative rigor by organizing cited studies according to thematic categories and explicitly identifying areas of agreement or disagreement among findings. Acknowledge the limitations of the cited studies, such as sample size, context, or disciplinary scope. Clearly state that the article’s objective is theoretical and integrative rather than to provide evidentiary proof, while ensuring that arguments remain well-supported.

**Balance and Justification of Conclusions: **The concluding section employs an optimistic and aspirational tone that is not fully supported by the available evidence. Expressions such as “revolutionary potential” and “authentic educational transformation” may exaggerate the current maturity of the evidence base.

 Recommendations: Revise the conclusions to more clearly differentiate between findings that are currently supported, those that appear promising, and those that remain conditional or speculative. Additionally, reiterate unresolved challenges as priorities for ongoing research and practice. It is also recommended to include a brief paragraph on the boundary conditions for transformation, outlining prerequisites such as teacher training, infrastructure, governance, and contextual factors.

 Overall recommendations: The manuscript is well-structured, timely, and theoretically informed, offering a valuable contribution to ongoing discussions on AI, gamification, and pedagogy. If the revisions outlined above are implemented, particularly those clarifying the distinction between evidence and advocacy, the manuscript will achieve a high standard of scientific clarity, balance, and conceptual rigor suitable for indexing.

Is the topic of the opinion article discussed accurately in the context of the current literature?

Partly

Are arguments sufficiently supported by evidence from the published literature?

Partly

Are all factual statements correct and adequately supported by citations?

Partly

Are the conclusions drawn balanced and justified on the basis of the presented arguments?

Partly

Reviewer Expertise:

AI in education

I confirm that I have read this submission and believe that I have an appropriate level of expertise to confirm that it is of an acceptable scientific standard, however I have significant reservations, as outlined above.

# Response

**Published:** 2026-04-06

**Authors:** Ana Dolores Vargas Sánchez

**Affiliations:** Cundinamarca, Universidad de La Sabana, Chia, Cundinamarca, Colombia

First, we want to thank the reviewer for all of their suggestions and help with the essay. In response to these suggestions, the paper was changed to include more clearly critical, mixed, and contextual views on how well gamification and artificial intelligence work in learning. New subsections were added: "Beyond optimistic perspectives: Rethinking the effectiveness of gamification and AI in learning" and "A critical reading of current evidence: points of agreement, gaps, and open questions." These sections bring together studies with different, null, or conditional results and look at things like how motivation changes over time, implementation failures, and how results can change depending on the situation. Moreover, the notion that the essay embodies a singular viewpoint within a continuous scholarly discourse, rather than a developing consensus, was substantiated.

 A more stringent match was also obtained between the levels of evidence and the language utilized. We changed strong or causally deterministic words like "significant," "radical," or "transformative" to more cautious words like "suggests," "indicates," or "has the potential to." It was made apparent which statements were normative and which were conceptual, and it was made explicit in each case whether the research listed were empirical, conceptual, or systematic reviews. To bolster the argumentation foundation, recent review papers were included, and it was clearly stated when general conclusions were backed by systematic or integrative evidence.

 Lastly, the thematic reorganization of the literature and the addition of a new table (Table 1) that separates empirical findings, emerging evidence, and conceptual propositions, as well as the main methodological limitations of each study, made the analysis more rigorous and structured. The conclusions were rewritten to make it apparent which findings are now supported, which are promising but not yet proven, and which are conditional or speculative. They also made it plain which difficulties still need to be solved and should be the focus of future study. A last paragraph was added to talk about the circumstances that need to be met for educational reform to work. This included things like teacher training, technical infrastructure, institutional governance, and implementation contexts.

# Reviewer report: Reviewer response for version 1

**DOI:** 10.5256/f1000research.189063.r426853

**Published:** 2025-11-20

**Authors:** Alexander Pfeiffer

**Affiliations:** 1 University for Continuing Education Krems, Krems, Krems, Austria

This opinion article by Barriga, Sánchez, and Vizarreta examines the convergence of AI and deep gamification within educational contexts. The authors argue that this synergy, when guided by a critical pedagogical framework, has the potential to foster genuine educational transformation. The paper presents AI not merely as a tool for efficiency but as a component of a critical pedagogy that places the student at the centre of learning. It explores how AI can enhance deep gamification to create personalized, adaptive, and meaningful learning experiences that promote intrinsic motivation and 21st-century skills. The authors provide a balanced perspective, discussing both the transformative potential of this integration and the significant ethical, technological, and pedagogical challenges that must be addressed, including data privacy, equity in access, and the crucial role of teacher training and autonomy.

 While the paper is a valuable contribution to the discussion already, the following minor revisions could enhance its clarity and impact. These are suggestions for improvement rather than mandatory corrections for scientific soundness.

 1. Provide a more explicit definition of "Deep Gamification" Early in the Manuscript: The distinction between deep and shallow gamification is central to the paper's thesis. While the authors explore this difference in the section "From motivation to transformation," the article would benefit from a concise, consolidated definition or a comparative table earlier on.

 2. Consolidate discussion of teacher competencies: The paper rightly emphasizes the crucial role of the teacher. The discussion of teacher training, autonomy, and pedagogical judgment appears in several sections. The authors could consider adding a short, dedicated subsection to consolidate the specific competencies teachers need to navigate this new landscape effectively. This would bring greater focus to one of the paper's most important practical implications.

Is the topic of the opinion article discussed accurately in the context of the current literature?

Yes

Are arguments sufficiently supported by evidence from the published literature?

Yes

Are all factual statements correct and adequately supported by citations?

Yes

Are the conclusions drawn balanced and justified on the basis of the presented arguments?

Yes

Reviewer Expertise:

Gamification, Serious Games, Game-based learning, AI, DLTs

I confirm that I have read this submission and believe that I have an appropriate level of expertise to confirm that it is of an acceptable scientific standard, however I have significant reservations, as outlined above.

# Response

**Published:** 2025-12-10

**Authors:** Ana Dolores Vargas Sánchez

**Affiliations:** Cundinamarca, Universidad de La Sabana, Chia, Cundinamarca, Colombia

We sincerely appreciate your feedback, which has contributed to improving the clarity and impact of the manuscript “Deep Gamification and Artificial Intelligence as Catalysts of Educational Transformation.” In response to your first suggestion, we have incorporated an explicit definition of “deep gamification” in the section “From motivation to transformation: deep gamification and AI in 21st century education” (page 12). Furthermore, we have added a conceptual clarification that differentiates deep gamification from AI-driven personalization, complemented by Figure 2 (page 13), which illustrates the main distinctions and their pedagogical implications.

 Regarding your recommendation to consolidate the discussion of teacher competencies, we have included a specific summary on pages 8–11, based on the UNESCO competency framework (2024). This section highlights the essential skills that teachers require to oversee AI systems, ensure their ethical use, and contextualize the generated content, thus reinforcing the practical dimension of the article.

 We believe these improvements directly address your feedback and strengthen the manuscript's structure, offering clearer definitions and a more teacher-centered approach. Our aim is for the article to provide not only theoretical value but also concrete tools for the responsible integration of AI and gamification in educational settings.

# Reviewer report: Reviewer response for version 1

**DOI:** 10.5256/f1000research.189063.r426859

**Published:** 2025-10-31

**Authors:** Rocsana Manea Tonis

**Affiliations:** 1 National University of Physical Education and Sports, Bucharest, Bucharest, Romania

The article presents a highly relevant and visionary argument that combines AI innovation with pedagogical humanism. It will appeal to audiences in educational technology, digital pedagogy, and critical studies. However, to reach scientific soundness, it needs clearer conceptual grounding, stronger methodological transparency, and tighter structure.

 Points That Must Be Addressed for Scientific Soundness

 - Add a short section describing the method of analysis (e.g., conceptual review, critical synthesis of literature). Optionally include a case example or framework diagram showing how AI supports deep gamification in real contexts.

 - Define precisely what constitutes “deep gamification” and how it differs from “AI-driven personalization.” Consider including a table summarizing differences and pedagogical implications.

 - Expand the section on teacher autonomy and ethical oversight. How do teachers mediate algorithmic bias? What competencies are required?

 - The article promotes AI-enhanced gamification while warning of over-dependence. A balanced framework reconciling these tensions is needed (e.g., “Pedagogical AI integration principles”).

 - Consolidate overlapping discussions (e.g., on 21st-century skills). Consider moving empirical examples into a distinct section (“Empirical Illustrations of AI–Gamification Synergy”).

 - Add at least one diagram or conceptual framework figure illustrating the relationship between AI, gamification, and pedagogy.

Is the topic of the opinion article discussed accurately in the context of the current literature?

Yes

Are arguments sufficiently supported by evidence from the published literature?

Yes

Are all factual statements correct and adequately supported by citations?

Yes

Are the conclusions drawn balanced and justified on the basis of the presented arguments?

Yes

Reviewer Expertise:

Cybermarketing - implementing AI, ML, blockchain

I confirm that I have read this submission and believe that I have an appropriate level of expertise to state that I do not consider it to be of an acceptable scientific standard, for reasons outlined above.

# Response

**Published:** 2025-12-10

**Authors:** Ana Dolores Vargas Sánchez

**Affiliations:** Cundinamarca, Universidad de La Sabana, Chia, Cundinamarca, Colombia

Your comments have greatly improved the scientific rigor and conceptual coherence of the manuscript "Deep Gamification and Artificial Intelligence as Catalysts of Educational Transformation." Thank you very much. As a result of your first suggestion, we have added a methodological section to the beginning of the article that explains the analytical approach we used, which was a conceptual review and critical synthesis of the literature. We also included a case study with a diagram showing how AI improves deep gamification in real-world educational settings.

 We also took your advice to make the definition of "deep gamification" clearer and to set it apart from "AI-driven personalization." To do this, we incorporated a lengthy explanation and a diagram that shows the distinctions in ideas and how they affect teaching. We also talked more about teacher autonomy and ethical supervision, including ways to reduce algorithmic bias and important skills based on the UNESCO framework (2024). This made sure that AI integration was done in a responsible and human-centered way.

 Finally, we have combined duplicate portions and created a balanced framework that balances the revolutionary power of AI with the dangers of relying too much on it. This framework is based on teaching concepts and international standards. It is also supported with conceptual diagrams that show how AI, gamification, and pedagogy are related. We think that with these changes, the paper fully responds to your comments and presents a clearer, more rigorous approach that follows best practices in education.
